# Abnormal Activation of BMP Signaling Causes Myopathy in *Fbn2* Null Mice

**DOI:** 10.1371/journal.pgen.1005340

**Published:** 2015-06-26

**Authors:** Gerhard Sengle, Valerie Carlberg, Sara F. Tufa, Noe L. Charbonneau, Silvia Smaldone, Eric J. Carlson, Francesco Ramirez, Douglas R. Keene, Lynn Y. Sakai

**Affiliations:** 1 Department of Biochemistry & Molecular Biology, Oregon Health & Science University, Portland, Oregon, United States of America; 2 Shriners Hospital for Children, Portland, Oregon, United States of America; 3 Department of Pharmacology and Systems Therapeutics, Mount Sinai School of Medicine, New York, New York, United States of America; The Jackson Laboratory, UNITED STATES

## Abstract

Fibrillins are large extracellular macromolecules that polymerize to form the backbone structure of connective tissue microfibrils. Mutations in the gene for fibrillin-1 cause the Marfan syndrome, while mutations in the gene for fibrillin-2 cause Congenital Contractural Arachnodactyly. Both are autosomal dominant disorders, and both disorders affect musculoskeletal tissues. Here we show that *Fbn2* null mice (on a 129/Sv background) are born with reduced muscle mass, abnormal muscle histology, and signs of activated BMP signaling in skeletal muscle. A delay in Myosin Heavy Chain 8, a perinatal myosin, was found in *Fbn2* null forelimb muscle tissue, consistent with the notion that muscle defects underlie forelimb contractures in these mice. In addition, white fat accumulated in the forelimbs during the early postnatal period. Adult *Fbn2* null mice are already known to demonstrate persistent muscle weakness. Here we measured elevated creatine kinase levels in adult *Fbn2* null mice, indicating ongoing cycles of muscle injury. On a C57Bl/6 background, *Fbn2* null mice showed severe defects in musculature, leading to neonatal death from respiratory failure. These new findings demonstrate that loss of fibrillin-2 results in phenotypes similar to those found in congenital muscular dystrophies and that *FBN2* should be considered as a candidate gene for recessive congenital muscular dystrophy. Both *in vivo* and *in vitro* evidence associated muscle abnormalities and accumulation of white fat in *Fbn2* null mice with abnormally activated BMP signaling. Genetic rescue of reduced muscle mass and accumulation of white fat in *Fbn2* null mice was accomplished by deleting a single allele of *Bmp7*. In contrast to other reports that activated BMP signaling leads to muscle hypertrophy, our findings demonstrate the exquisite sensitivity of BMP signaling to the fibrillin-2 extracellular environment during early postnatal muscle development. New evidence presented here suggests that fibrillin-2 can sequester BMP complexes in a latent state.

## Author Summary

New strategies for treating congenital muscular dystrophies are needed. Current treatments are limited and aim to prolong ambulation and survival. Since most of the genes responsible for congenital muscular dystrophies are still unknown, elucidation of these genes may provide new insights that can lead to novel treatments. Fibrillin-2 null mice are born with myopathy and contractures and demonstrate accumulation of white fat during the early postnatal period. Both the histological features of myopathy and the accumulation of fat are rescued by inhibiting BMP signaling. Results indicate that *FBN2* is a candidate gene for congenital muscular dystrophy and that strategies aimed at inhibition of abnormal BMP signaling may be applicable to muscular dystrophies. Furthermore, results reveal the importance of extracellular control of BMP signaling in skeletal muscle.

## Introduction

The fibrillins are large structural macromolecules that are important constituents of all connective tissues. Fibrillins form microfibrils [1–3], and they target growth factors to the extracellular space [4–6]. In humans, there are 3 fibrillin genes, whereas in mouse, the gene for fibrillin-3 was lost due to chromosomal rearrangement [7]. Expression of genes for fibrillin-2 and -3 is limited largely to fetal development [7,8]. However, fibrillin-2 protein remains within the tissue microfibrils in postnatal tissues [9].

Mutations in the gene for fibrillin-2 (*FBN2*) cause congenital contractural arachnodactyly (CCA) or Beals syndrome (now designated as Distal Arthrogryposis, Type 9, OMIM #121050), a dominantly inherited disorder of connective tissue. Muscle weakness was noted in some cases of CCA [10], but it has not been emphasized (OMIM #121050). Characteristic features include contractures of the large and small joints, arachnodactyly, scoliosis, and crumpled ears. In contrast to humans with a heterozygous mutation, *Fbn2* null mice are born with syndactyly and contractures [11]. They also have persistent impairment of locomotory function in their hindlimbs which is not due to defects in the peripheral nervous system [12]. Similar muscle weakness has been reported in the *Fbn2* mutant “Mariusz” mouse, identified as part of a large-scale N-ethyl-N-nitrosourea (ENU) mutagenesis screen [13].

Abnormal activation of TGFβ signaling was found to contribute to several features of the Marfan syndrome (MFS), a dominantly inherited disorder caused by mutations in the gene for fibrillin-1 (*FBN1*). Hallmark features of MFS include aortic aneurysm and dissection; tall stature, arachnodactyly, scoliosis, and loose joints; and ectopia lentis. In addition, pneumothorax and skeletal muscle hypoplasia are also characteristic of MFS. Activated TGFβ signaling was found in the lung [14], aorta [15], and skeletal muscle [16] of Marfan mice. Loss of fibrillin-2 in mice was associated with syndactyly and loss of BMP signaling during limb bud patterning [11]. Dysregulated growth factor signaling has not been found in musculoskeletal tissues associated with contractures or hypermobility in CCA or MFS.

Scoliosis and arachnodactyly are features of both CCA and MFS. However, instead of the hypermobile joints characteristic of MFS, there are contractures of the large and small joints in CCA. Certain *FBN1* mutations cause syndromes with musculoskeletal features that are the opposite of MFS (short stature acromelic dysplasias such as Weill-Marchesani syndrome (WMS) [17,18] and geleophysic dysplasia or acromicric dysplasia [19]). Skeletal muscle mass in MFS is decreased, whereas hypermuscularity is a feature of WMS. The conundrum posed by opposing clinical features resulting from mutations in the same gene (*FBN1*) or in related genes (*FBN1* or *FBN2*) may be explained by differential effects of mutations on the activation or inhibition of growth factor signaling.

Although the genetic data demonstrate differential, or in some cases even opposite, phenotypic effects of fibrillin mutations, knowledge of tissue-specific mechanisms involved in differential effects on growth factor signaling by fibrillins is scant. In skeletal muscle, a role for fibrillin-1 in regeneration was attributed to the control of TGFβ signaling by fibrillin-1 [16]. Roles for fibrillin-2 in skeletal muscle development and disease have not been studied, especially within the context of growth factor signaling. The investigations presented here address this unexplored area.

Results show that loss of fibrillin-2 is accompanied by a decrease in muscle mass and an increase in white fat during the early postnatal period. *In vitro* and *in vivo* analyses demonstrate that abnormal activation of BMP signaling contributes to this myopathic phenotype. These results are somewhat surprising, since recent studies have shown that BMP signaling promotes muscle hypertrophy and also protects muscles from denervation-induced wasting [20,21]. An additional recent study indicates that loss of BMP signaling promotes intramuscular adipogenesis [22]. In contrast to these recent studies, which rely on the manipulation of cellular components of the BMP signaling pathway, our study focuses on the role of extracellular modulation of BMP signaling. By changing the extracellular environment for BMP signaling, we show that BMP signaling is context dependent.

Mutations in extracellular structural macromolecules can cause congenital forms of muscular dystrophy. The congenital muscular dystrophies are a heterogeneous group of disorders that are identified at birth or within the first few years of life. Typical clinical features include hypotonia, delayed motor development, and progressive muscle weakness. The congenital muscular dystrophies caused by merosin (laminin-2) deficiency or defects in collagen VI appear early and are usually severe. Molecular diagnostics over the last two decades have advanced understanding of the muscular dystrophies, but treatment strategies remain limited. Conventional treatments are physical therapy and corticosteroids, and these are aimed at prolonging ambulation and survival.

It has been estimated that only half of the congenital muscular dystrophies can be ascribed to known defects [23]. Therefore, it remains important to identify additional muscular dystrophy disease genes, especially if these might lead to novel pathogenetic mechanisms common to at least some of the muscular dystrophies. Common pathogenetic targets might then lead to new opportunities to develop better treatments. Here we present novel data that support the candidacy of *FBN2* as a possible congenital muscular dystrophy gene as well as a potential pathogenetic pathway amenable to treatment protocols.

## Results

### Forelimb contractures in newborn Fbn2 null mice

**Fig 1:**
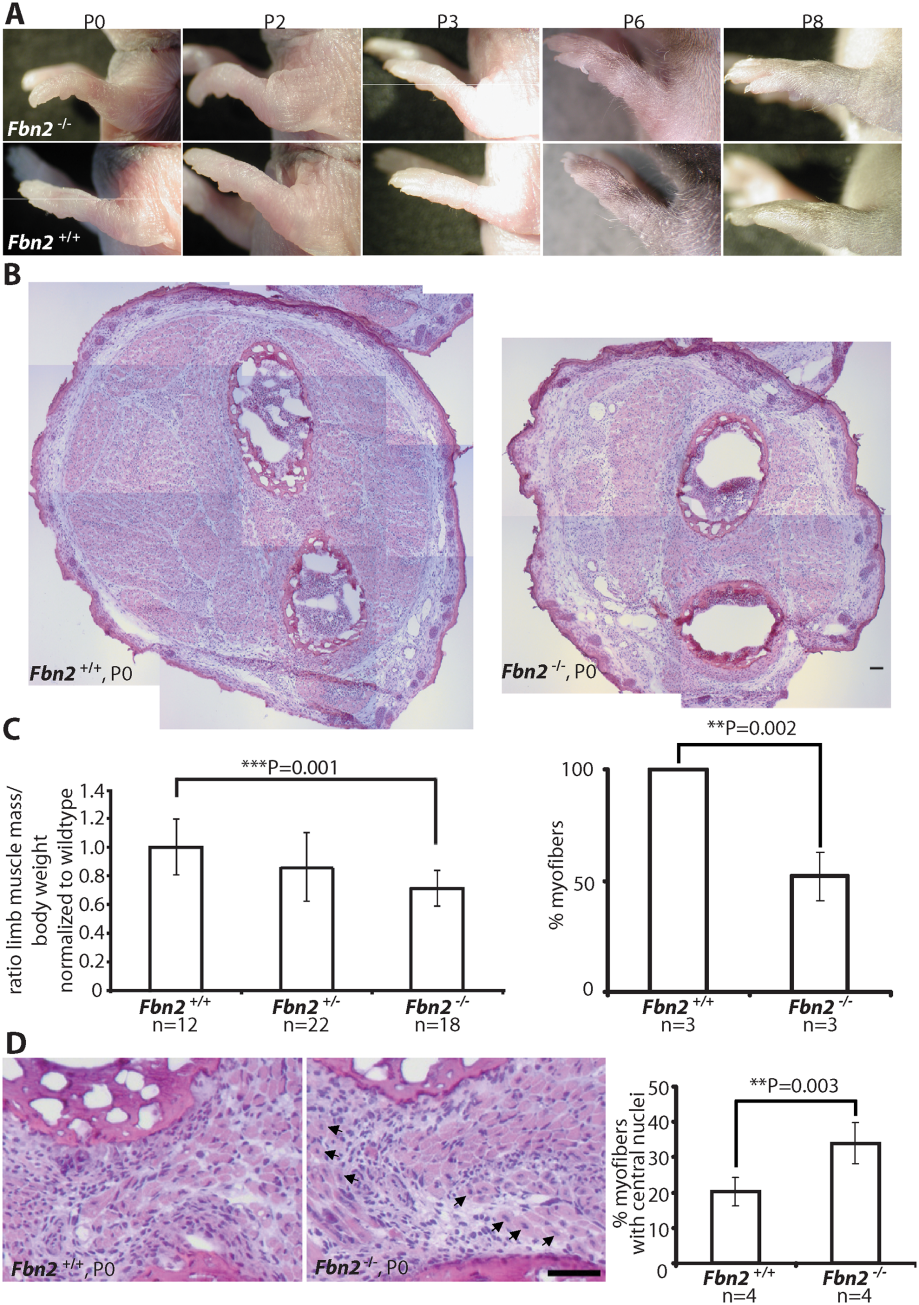
Forelimb contractures, reduced muscle mass and altered muscle morphology in P0 *Fbn2* null mice. **(A)** *Fbn2* null forelimb contractures from P0 to P8. **(B)** Representative H&E stained P0 forearm cross sections from the same approximate position in the forearm. The cross sections are composites of multiple micrographs. **(C) Left:** Quantitation of total forearm muscle mass dissected from *Fbn2* ^*+/+*^, *Fbn2* ^+/-^, and *Fbn2* ^-/-^ animals. The ratio of total muscle mass/total body weight was normalized to wildtype. N = the number of animals used. **Right:** 50% reduction of myofibers, counted on all micrographs forming composite cross sections of *Fbn2* null forearm muscle compared to wildtype. N = the number of animals used. **(D)** H&E stained sections **(left)** of *Fbn2* null forearm muscle at P0 with increased numbers of myofibers with central nuclei (arrows) compared to wildtype and quantitation of myofibers with central nuclei **(right)**. When myofibers in all muscle groups were counted for Fig 1C, a second count was performed for myofibers with central nuclei. Numbers were added and divided by the total number of myofibers (obtained for 1C) to obtain percentage with central nuclei. N = the number of animals used. Error bars indicate mean ± SD, and asterisks indicate statistically significant differences (p < 0.05) between genotypes. Bars = 50 μm.

On 129/Sv background, *Fbn2* null mice are viable and fertile, but are born with contractures of their forelimbs which disappear within the first week of postnatal life [11]. Because of the similarity between the contractures found in *Fbn2* null mice and in humans with CCA, *Fbn2* null mice are a model for CCA. Daily inspection of the *Fbn2* null forelimbs during the first week of life revealed that the contractures were most severe between birth and P2 and slowly resolved between P3 and P8 (Fig 1A).

### Skeletal muscle abnormalities in early postnatal Fbn2 null mice

Since contractures are a common feature of congenital muscular dystrophies, we analyzed *Fbn2* null forelimb muscle at birth (P0 or P1), when contractures were most severe, and at P8, when contractures had resolved. At P0, comparable cross sections of forearms demonstrated strikingly reduced areas of skeletal muscle in *Fbn2* null mice when compared to wildtype mice (Fig 1B). In order to quantitate the reduction in muscle mass, forearm muscle tissue between the wrist and elbow was dissected and weighed, and the weight of the forearm muscle was normalized to the total body weight. Results showed that the total forearm muscle mass in *Fbn2* null mice was reduced by 28% when compared to wildtype (p = 0.001) (Fig 1C, left). In addition, when myofibers were counted on comparable sections, the number of myofibers in *Fbn2* null muscle areas was reduced by 50% (p = 0.002) (Fig 1C, right). H&E staining also showed significant alterations in muscle architecture measured by increased numbers (1.7 fold, p = 0.003) of myofibers with centrally located nuclei in *Fbn2* null muscle compared to wildtype (Fig 1D).

**Fig 2:**
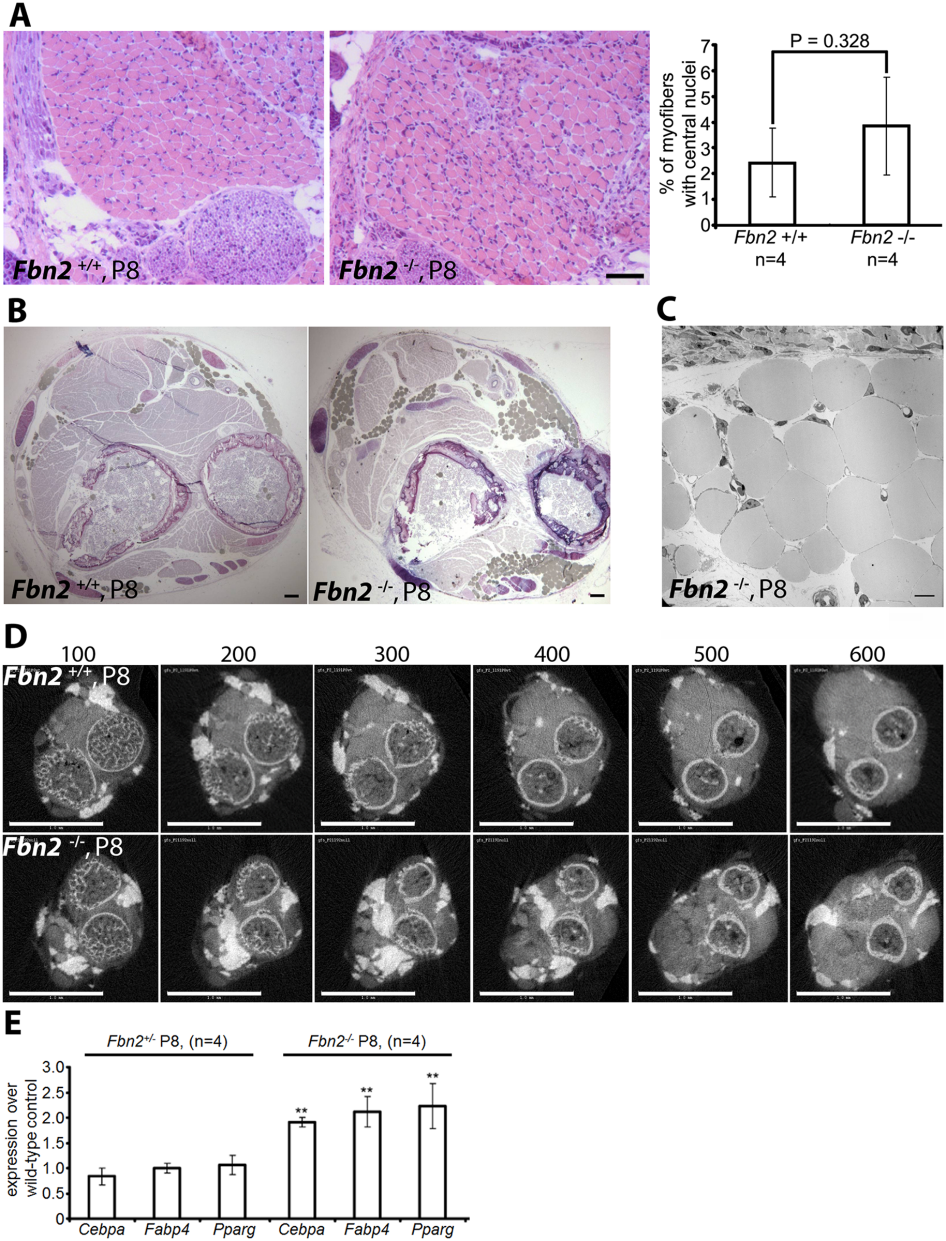
Muscle architecture and forelimb fat at P8. **(A)** H&E stained cross sections of wildtype and *Fbn2* null forearms at P8 **(left)** and quantitation of percentages of myofibers with central nuclei **(right)**. Quantitation was performed as in 1C and 1D. **(B)** Toluidine Blue stained cross sections of P8 wildtype and *Fbn2* null forearms showing accumulation of fat droplets (filled grey spheres) in the null forearm. Bars = 50 μm in **(A)** and **(B)**. **(C)** Transmission electron microscopy of fat droplets in P8 *Fbn2* null forearm muscle. The micrograph was taken from a region containing fat droplets in the block shown in (**B**). Bar = 10 μm. **(D)** A comparable series of 3 μm thick sections of wildtype and *Fbn2* null forearms generated by micro-CT (sections numbered above each panel). Fat and bone appear white; fat is solid white, while bone includes grey space within the circular bone collar. Bars = 1 mm. **(E)** qPCR anaysis of fat specific markers in *Fbn2* heterozygous and null mice compared to wildtype littermates.

By P8, when contractures were apparently resolved, H&E staining of *Fbn2* null forearm cross sections showed that muscle architecture had improved. The number of myofibers with centrally located nuclei was no longer significantly greater than in wildtype (Fig 2A, right). However, toluidine blue staining on comparable cross sections showed increased fat deposition in the forearms of *Fbn2* null mice (Fig 2B). Transmission electron microscopy of fat droplets identified them as white adipocytes—cells containing large lipid droplets with eccentric flattened nuclei (Fig 2C). Skinned and strongly fixed P8 forearms were infiltrated with OsO_4_ to allow the visualization of both fat and bone as white matter by micro-computed tomography (μCT) (Fig 2D) (16). A series of digital sections demonstrated increased depositions of fat throughout most of the forearm muscle from the wrist to the elbow (Fig 2D). In wildtype forearms, fat was located at the periphery of muscles; in contrast, fat in *Fbn2* null forearms was found between muscle and bone and infiltrating muscle bundles (Fig 2D). Forearm muscle was studied in detail, in order to associate muscle abnormalities with contractures. However, whole body scans with μCT were performed using P5 littermates. Hindlimb muscles also showed reduced muscle mass and abnormal fat deposition (S1 Fig).

Quantitative real-time PCR (qPCR) analysis of P8 forearm muscle showed a significant twofold increase of fat specific markers such as *Cebpa*, *Fabp4*, and *Pparg* in *Fbn2* null muscle compared to heterozygous or wildtype (Fig 2E). These results support the morphological analyses and μCT quantitation of increased fat deposition in the forearm. qPCR analysis of *Fbn1* and *Fbn2* expression levels showed no abnormal increase in expression of *Fbn1* or *Fbn2* to compensate for loss of *Fbn2* (S2 Fig).

### Elevated creatine kinase activity in blood from Fbn2 null mice

Reduced muscle mass, replacement of muscle with fat, centrally localized nuclei in skeletal muscle, and contractures are features associated with muscular dystrophy. Muscle weakness, including grip strength, hindlimb clasping, and poor wire maneuvering, was shown in adult “Mariusz” mice, which harbor an ENU-induced *Fbn2* mutation [12]. “Mariusz” mice were generated on a Balb/c X C3H background. Hindlimb clasping and poor hindlimb gait are also characteristic of adult *Fbn2* null mice on a 129/Sv background, implying that muscle weakness is a persistent phenotype in this background as well. Since elevated creatine kinase activity is used as a sign of muscle damage, blood samples from *Fbn2* null and wildtype mice were tested for creatine kinase activity. Age-matched (from 2 months to 1 year) wildtype and *Fbn2* null mice were tested. Creatine kinase activity was measured in blood at 6 time points, and the–fold change between wildtype and null mice was averaged. Creatine kinase activity in adult *Fbn2* null mice was 2.7 fold (+/- 1.4) times greater than in wildtype mice (Table 1). These results are consistent with the persistent muscle weakness observed in adult *Fbn2* mice.

**Table 1:** Creatine kinase activities in blood samples from wildtype and *Fbn2* null mice. Mean +/- standard deviation for a given genotype and age is shown, except where only one animal was tested. N = number of animals tested. Units are nmol NADH/min/ml.

Age	WT	Null	Fold increase
2 months	558 +/- 78 (n = 2)	1433 (n = 1)	2.6
3 months	275 (n = 1)	618 +/- 91 (n = 2)	2.2
4 months	237 +/- 118 (n = 2)	1268 (n = 1)	5.4
5 months	62 +/- 11.5 (n = 2)	226 +/- 105 (n = 3)	3.6
7 months	360 +/- 185 (n = 2)	628 +/- 385 (n = 2)	1.7
1 year	206 (n = 1)	307 (n = 1)	1.5

### Severe congenital myopathy in Fbn2 null mice on a C57/Bl6 background

**Fig 3:**
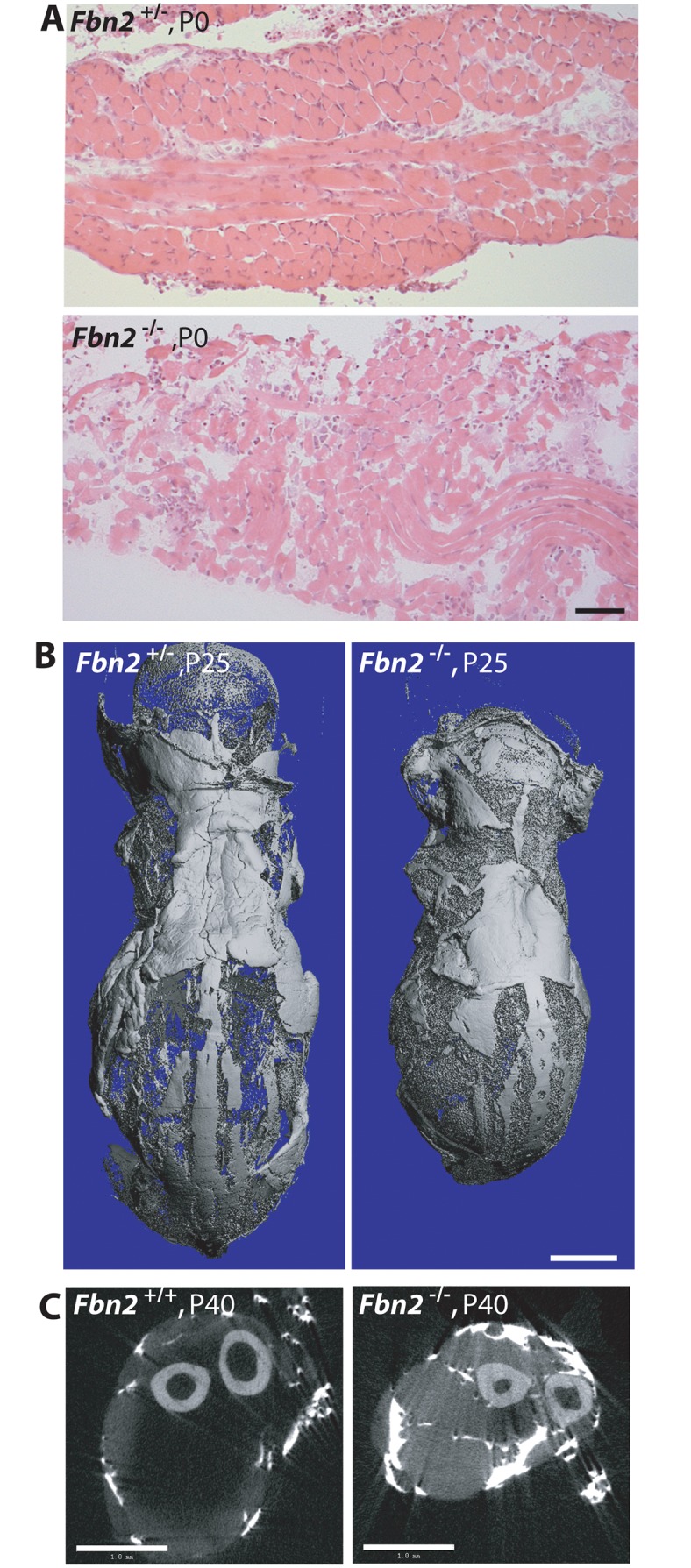
Muscle and fat in *Fbn2* null mice on a C57/Bl6 background. **(A)** H & E stained sections of diaphragm muscle from P0 *Fbn2* heterozygous and null littermates. Bar = 50μm. **(B**) μCT images of skinned and osmicated P25 *Fbn2* heterozygous and null littermates showing comparable subcutaneous fat. Bar = 5 mm. **(C)** μCT section of P40 wildtype and *Fbn2* null forearms. Bar = 1 mm.

To evaluate the influence of genetic background, we crossed the *Fbn2* null allele from the 129/Sv mouse background onto a C57/Bl6 background by breeding *Fbn2* ^*+/-*^ mice to wildtype C57/Bl6 mice. After six generations on C57/Bl6, *Fbn2* ^*+/-*^ mice were bred together to generate *Fbn2* null progeny. Of 47 pups (7 litters), 12 were null for *Fbn2*. Six of the 12 *Fbn2* null pups died in the perinatal period. Some of these pups were found alive at birth, but they died soon after birth. Necropsy showed normal lungs and heart with no blood in the chest cavity. Central cyanosis suggested that the *Fbn2* null pups died from hypoventilation. Gross observation of the diaphragm at P0, which looked thin and frail, indicated reduced muscle mass, and H&E staining showed poor muscle architecture (Fig 3A). Two *Fbn2* null mice lived to P25 and P40, when they were sacrificed and analyzed by μCT. These mice were clearly smaller than their wildtype littermates (Fig 3B and 3C). μCT showed that accumulation of fat was not present in the subcutaneous tissues (Fig 3B) but was rather limited to the forelimb connective tissue. Hence, poor muscle architecture and the specific replacement of muscle tissue with fat were similar in *Fbn2* null mice on 129/Sv and C57/Bl6 backgrounds, but the myopathy was less severe on the 129/Sv background. These results indicate the importance of genetic modifiers on the severity of *Fbn2* myopathy.

Results obtained after 6 generations on C57/Bl6 were not uniform. In addition, the possibility remained that further backcrossing into the pure C57/Bl6 background might lead to fetal lethality. After 9 generations on C57/Bl6, 4 litters with 21 total pups contained 4 *Fbn2* null pups, each of which died at P0. These results indicate that, when the *Fbn2* null allele is on a pure C57/Bl6 background, death of *Fbn2* null mice will occur shortly after birth.

### A delay in skeletal muscle differentiation associated with loss of fibrillin-2

**Fig 4:**
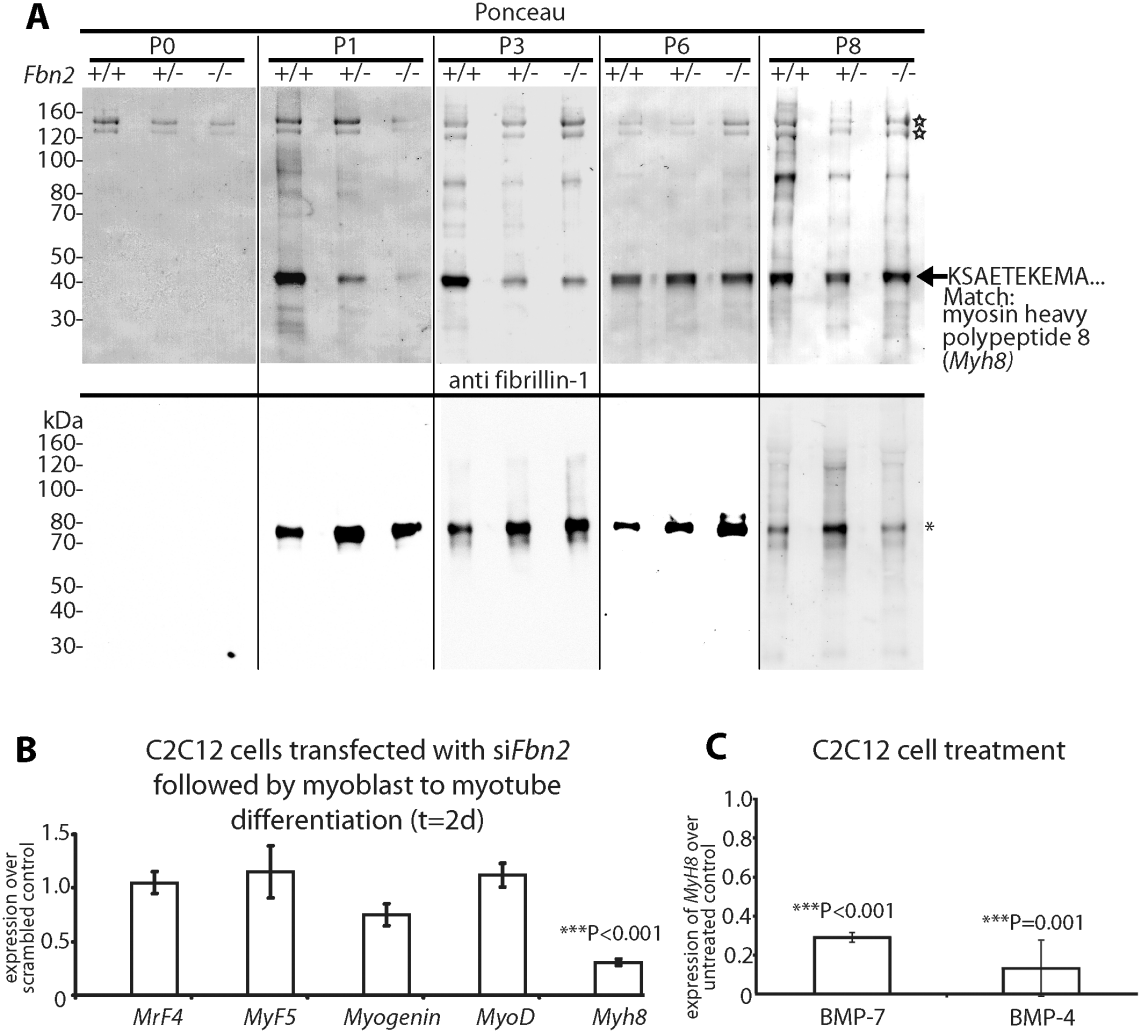
Myosin heavy chain 8, a perinatal myosin, in *Fbn2* null forelimb muscle and in C2C12 cultures. **(A)** Pepsin-resistant fragments extracted from P0 –P8 forelimb muscles, visualized after Ponceau staining (top) and immunoblotting with anti-fibrillin-1 (bottom). Asterisk indicates pepsin-resistant fibrillin-1 fragment. Open stars mark pepsin-resistant bands that are likely to be collagen I. Myosin heavy chain 8 was identified by N-terminal Edman sequencing. **(B)** Expression of *Myh8* during C2C12 myoblast to myotube differentiation while *Fbn2* was knocked down by RNAi. **(C)** Expression of *Myh8* in C2C12 cells treated with 100 ng/ml BMP-7 or 50 ng/ml BMP-4. Results in (B) and (C) were obtained from three independent experiments, and each experiment was performed in triplicates. Error bars indicate mean ± SD.

To assess possible effects on fibrillin-1, forearm muscles from *Fbn2* null and wildtype mice were dissected and digested with pepsin, following an established protocol [24], and pepsin-resistant fibrillin-1 fragments were analyzed over the first 8 days of postnatal life. We identified a pepsin-resistant fragment of fibrillin-1 with a *M*_*r*_ of 70 kDa (Fig 4A) similar to previously characterized fibrillin-1 fragments from human amnion [24]. At P0, when contractures were severe and muscle mass was reduced, no pepsin-resistant fibrillin-1 fragments were detected. However, from P1 to P8, fibrillin-1 bands (asterisk) of equal intensities were detected in wildtype, *Fbn2* ^+/-^, *Fbn2* ^*-/-*^ muscle, indicating that the amount of fibrillin-1 in forearm muscle tissue was not significantly altered by *Fbn2* genotype (consistent with qPCR data shown in S2 Fig). Since no pepsin-resistant fibrillin-1 fragments were identified in P0 tissue digests, fibrillin-1 microfibrils may undergo maturation processes after birth that are necessary to stabilize certain fibrillin-1 peptides to pepsin digestion. As a positive control for the pepsin digestions, Ponceau staining showed two bands (open stars), most likely the α1 and α2 chains of type I collagen, that were present in all extracts from P0 to P8.

The Ponceau staining also revealed a pepsin-resistant band around 40 kDa (Fig 4A). Surprisingly, the intensity of this band correlated with the *Fbn2* genotype when contractures were severe (P1-P3). Quantitation of this band in P1 and P3 *Fbn2* mutants compared to wildtype was performed, with normalization relative to the pepsin-resistant collagen bands. These data showed a significant reduction in both heterozygous and null forearms compared to wildtype (S3 Fig). N-terminal sequence analysis identified this band as myosin heavy chain polypeptide 8 (*Myh8*), a perinatal myosin present in skeletal muscle. To test whether loss of fibrillin-2 leads to reduced expression of *Myh8*, RNAi mediated knockdown of *Fbn2* was performed in C2C12 cells during myoblast to myotube differentiation. After 2 days, knockdown of *Fbn2* resulted in downregulation of *Myh8* while other myogenic factors like *Myogenin* and *MyoD* were not affected (Fig 4B). Similarly, knockdown of *Fbn2* had no effect on the expression of *Fbn1* (S4 Fig).

Taking all the data together, we conclude that severe contractures in *Fbn2* null mice are associated with reduced skeletal muscle mass, abnormal muscle histology, and a delay in expression of the perinatal *Myh8*. Interestingly, mutations in *MYH8* cause the Trismus-pseudocamptodactyly syndrome (a Carney complex variant) in humans, characterized by contractures in hands and feet [25]. Therefore, contractures in the *Fbn2* null mice may be caused by a delay in muscle differentiation.

BMP signaling is thought to cause a delay in muscle differentiation [26]. Hence, we tested whether increased BMP activity has an effect on *Myh8* expression. When C2C12 cells were utilized, *Myh8* expression was 3- to 10-fold downregulated after 24 hours of treatment with BMP-4 or BMP-7 (Fig 4C). This is consistent with previous observations that BMP treatment of C2C12 cells inhibited the formation of multinucleated myotubes and resulted in downregulation of myosin heavy chain expression [26]. In addition, these results suggest that BMP treatment of C2C12 cells has a similar negative effect on *Myh8* expression as RNAi-mediated knock down of *Fbn2* during myoblast to myotube formation.

### Activated BMP signaling in Fbn2 null muscle

**Fig 5:**
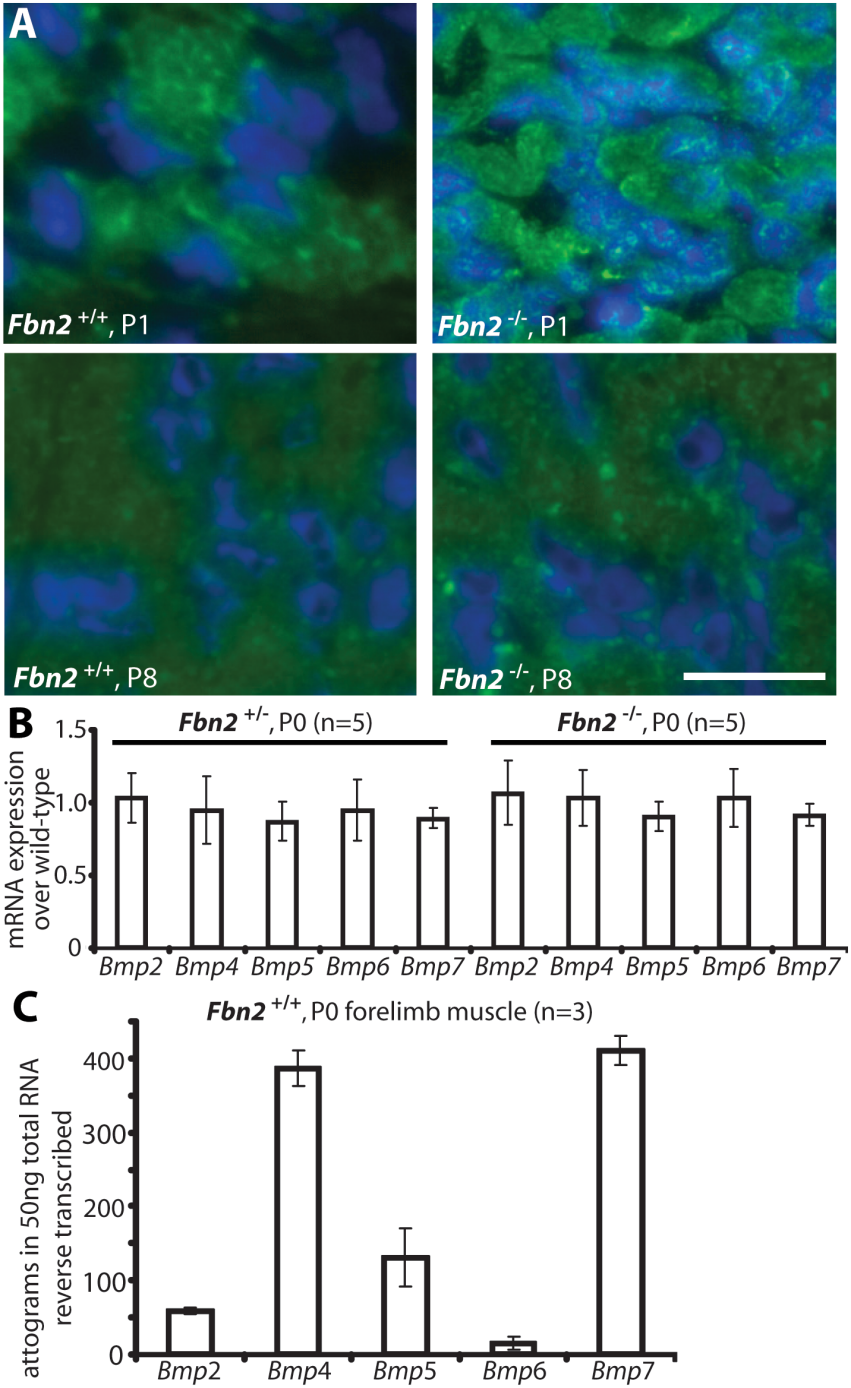
BMP signaling in wildtype and *Fbn2* null forearm muscle. **(A)** Phosphosmad 1/5/8 immunofluorescence signals (green dots) within DAPI blue-stained nuclei of *Fbn2* null muscle compared to wildtype at P1 and P8. **(B)** *BMP* mRNA expression levels in P0 *Fbn2* heterozygous and null muscle compared to wildtype. **(C)** Absolute expression levels of *Bmp* mRNA in P0 wildtype forearm muscles. N = the number of animals analyzed. Error bars indicate mean ± SD. Bars = 50 μm.

We therefore hypothesized that the increased numbers of muscle cells with centrally located nuclei in *Fbn2* null forearms were due to a delay in differentiation caused by activation of BMP signaling. To test this hypothesis, we analyzed cross sections of *Fbn2* null and wildtype forearms by immunofluorescence using antibodies against phospho-Smad (pSmad) 1/5/8 and pSmad 2/3, respective downstream mediators of BMP and TGFβ signaling. At early postnatal time points when contractures are severe, we detected abundant signals for pSmad 1/5/8 in *Fbn2* null, but not in wildtype, muscles (Fig 5A, upper panel). At P8, when contractures are resolved, pSmad 1/5/8 signals in *Fbn2* null forearm muscles were no longer detectable. In support of positive pSmad 1/5/8 staining, qPCR results for *Id1*, a BMP-responsive gene, showed a two-fold increase in P0 *Fbn2* null forearm extracts compared to wildtype (S5 Fig).

To exclude that activation of BMP signaling was due to upregulation of BMP expression, we measured levels of BMP mRNAs in *Fbn2* ^*+/-*^, *Fbn2* ^-/-^, and wildtype forearms by qPCR, but could not detect any significant differences in the mutants, when compared to wildtype (Fig 5B). We also used qPCR and external template standards of known concentrations for each *Bmp* qPCR primer set, in order to determine which of the *Bmp*s are most highly expressed in skeletal muscle. *Bmp4* and *7* had the highest expression in mouse forearm muscles at P0 (Fig 5C).

### Manipulation of BMP signaling in organ cultures of forearms

**Fig 6:**
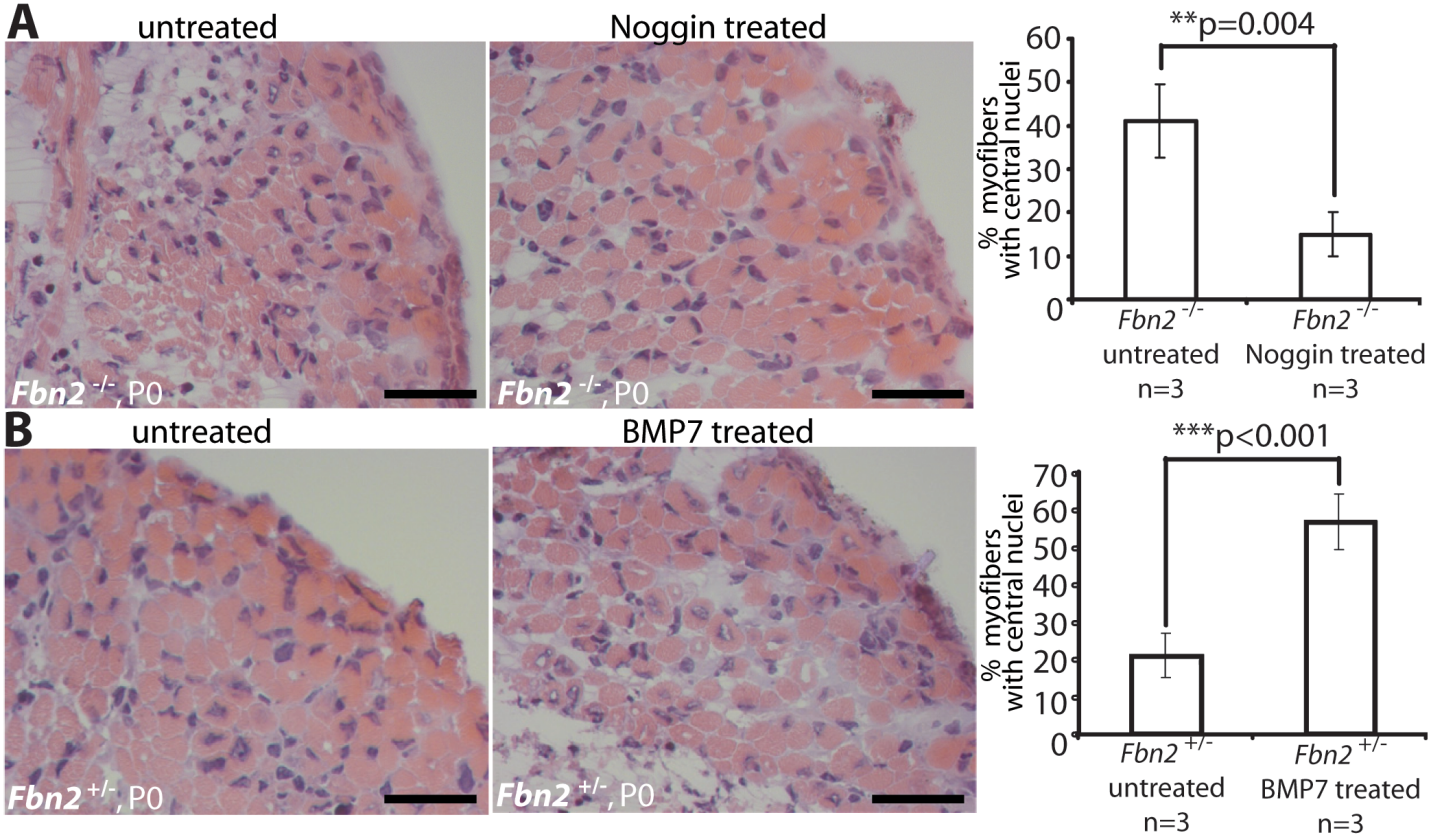
Limb organ cultures of heterozygous and *Fbn2* null forearm muscles. **(A)** H & E sections of *Fbn2* null muscle cultured with or without Noggin **(left)** and quantitation of myofibers with central nuclei **(right)**. **(B)** H & E sections of control forearms (*Fbn2* ^+/-^) cultured with or without BMP-7 **(left)** and quantitation of myofibers with central nuclei **(right)**. Sections from forelimbs were all taken from approximately the same plane in the forelimb. Five different sections from comparable areas in each forelimb were analyzed and counted. Error bars indicate mean ± SD, and asterisks indicate statistically significant differences (p < 0.05) between genotypes. Bars = 50 μm.

To test the hypothesis that activated BMP signaling causes the muscle phenotype observed in *Fbn2* null forelimbs, we treated null and wildtype forelimbs in organ culture with Noggin, a BMP inhibitor. Exogenous Noggin was titrated (200–800 ng/ml), and the number of myofibers with central nuclei was counted. Multiple fields from different H&E stained sections were counted, and the numbers were graphed as percentage of the total number of myofibers. Noggin treatment at 6 nM (200 ng/ml) for 2 days in organ culture was sufficient to significantly improve the muscle architecture, measured as the decrease of myofibers with central nuclei from 42 to 14% (p = 0,004; Fig 6A). In contrast, when control *Fbn2* ^+/-^ forelimbs were either treated with 6 nM (600 ng/ml) exogenous BMP-7 complex or were untreated for 2 days in organ culture, a significant increase of myofibers with central nuclei (from 20% in the untreated forelimb to almost 60% in the treated forelimb, p<0.001) was observed (Fig 6B).

### Genetic rescue of Fbn2 null myopathy

**Fig 7:**
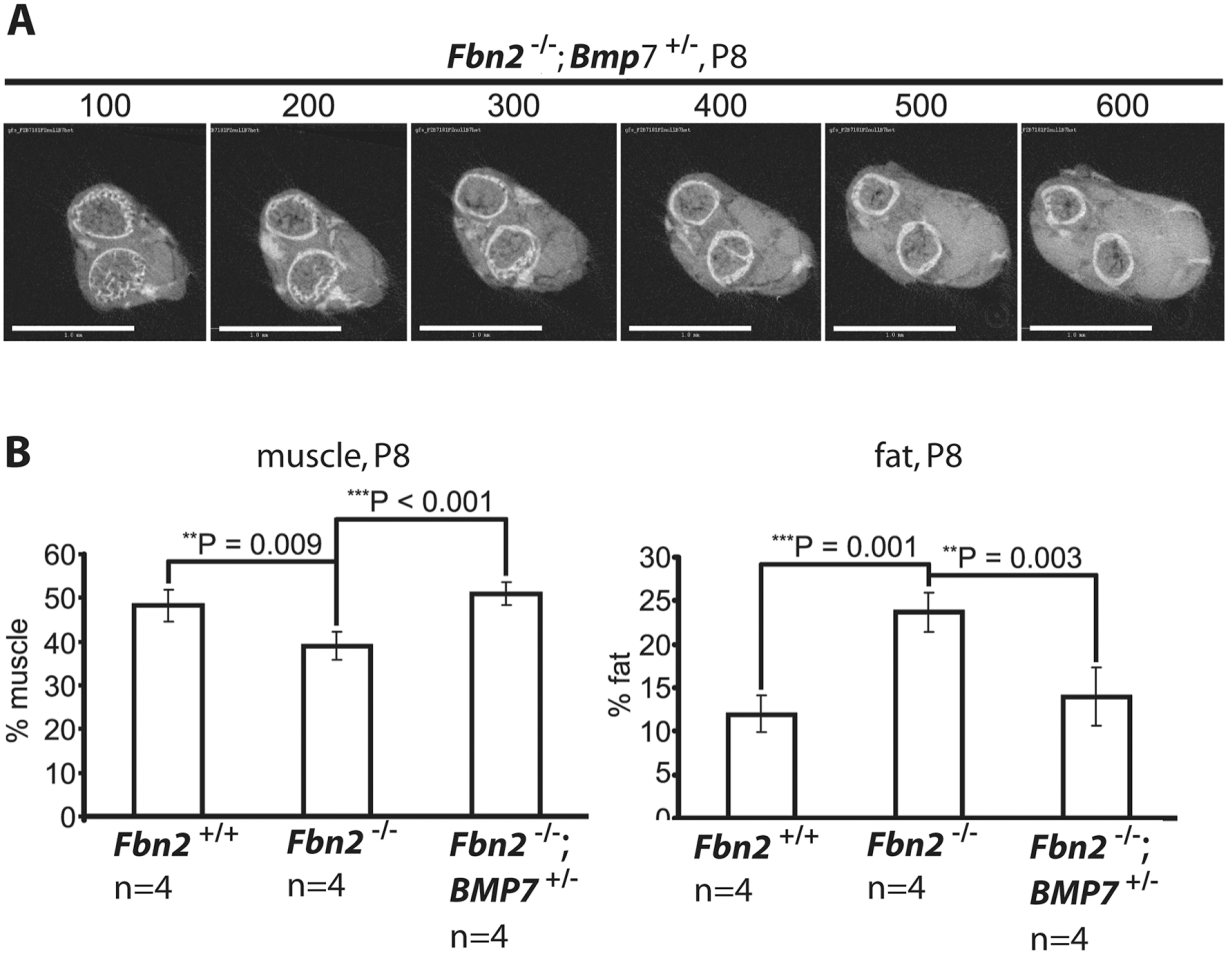
Effects of genetic ablation of one allele of *Bmp7* on *Fbn2* null forearm muscle and fat. **(A)** Series of sections (numbered at the top) generated by micro-CT of forearms from *Fbn2* ^-/-^;*Bmp*7^+/-^ mice (compare with Fig 2D). **(B)** Quantitation of percentages of muscle and fat across the genotypes. Error bars indicate mean ± SD, and asterisks indicate statistically significant differences (p < 0.05) between genotypes. Bars = 1 mm.

Since *Bmp7* was highly expressed in P0 mouse forelimb muscle and since addition of BMP-7 to forelimb organ cultures resulted in an increase in the numbers of myofibers with centrally localized nuclei, we hypothesized that lowering the expression level of *Bmp7* should have beneficial effects on the *Fbn2* null myopathy. To test this hypothesis, *Fbn2* ^+/-^ mice were crossed with *Bmp*7^+/-^ mice [27] to generate *Fbn2* ^+/-^; *Bmp7* ^+/-^ double heterozygous mice. *Fbn2* ^+/-^; *Bmp7* ^+/-^ mice were then crossed with each other to generate *Fbn2* ^-/-^; *Bmp7* ^+/-^ mice. Analysis and quantitation of forelimb muscle and fat was performed by μCT, after fixation and incubation of limbs with OsO_4_. A series of digital sections from *Fbn2* ^+/-^; *Bmp7* ^+/-^ forelimb is shown in Fig 7A. The percentages of fat on comparable μCT sections of *Fbn2* null and wildtype littermates were quantitated relative to the areas of bone and muscle. Fat in *Fbn2* null forearms was significantly increased by two-fold over wildtype (p = 0.003; Fig 7B). Analysis of *Fbn2* ^-/-^; *Bmp7* ^+/-^ forelimbs showed that the amount of fat and muscle on serial digital cross sections returned to normal wildtype levels (Fig 7B). This genetic approach provided further evidence that activated BMP signaling caused the *Fbn2* null myopathy, including both the reduction in muscle mass and the increase in fat that infiltrates the forelimb muscle.

### Control of BMP activity by fibrillin

It was previously shown that loss of fibrillin-2 results in syndactyly, likely due to a loss of BMP activity in the interdigital space [11]. In the developing interdigital space, loss of fibrillin-2 is accompanied by a loss of fibrillin-1 (S6 Fig). However, in early postnatal forelimb muscle, both fibrillins are detectable by immunostaining, and loss of fibrillin-2 is not accompanied by any substantial loss or redistribution of fibrillin-1 (S7 Fig). Moreover, loss of fibrillin-2 in the forelimb is accompanied by activation of BMP signaling (Fig 5A). Therefore, control of BMP activity by fibrillin cannot be explained by the simple presence or absence of fibrillin.

**Fig 8:**
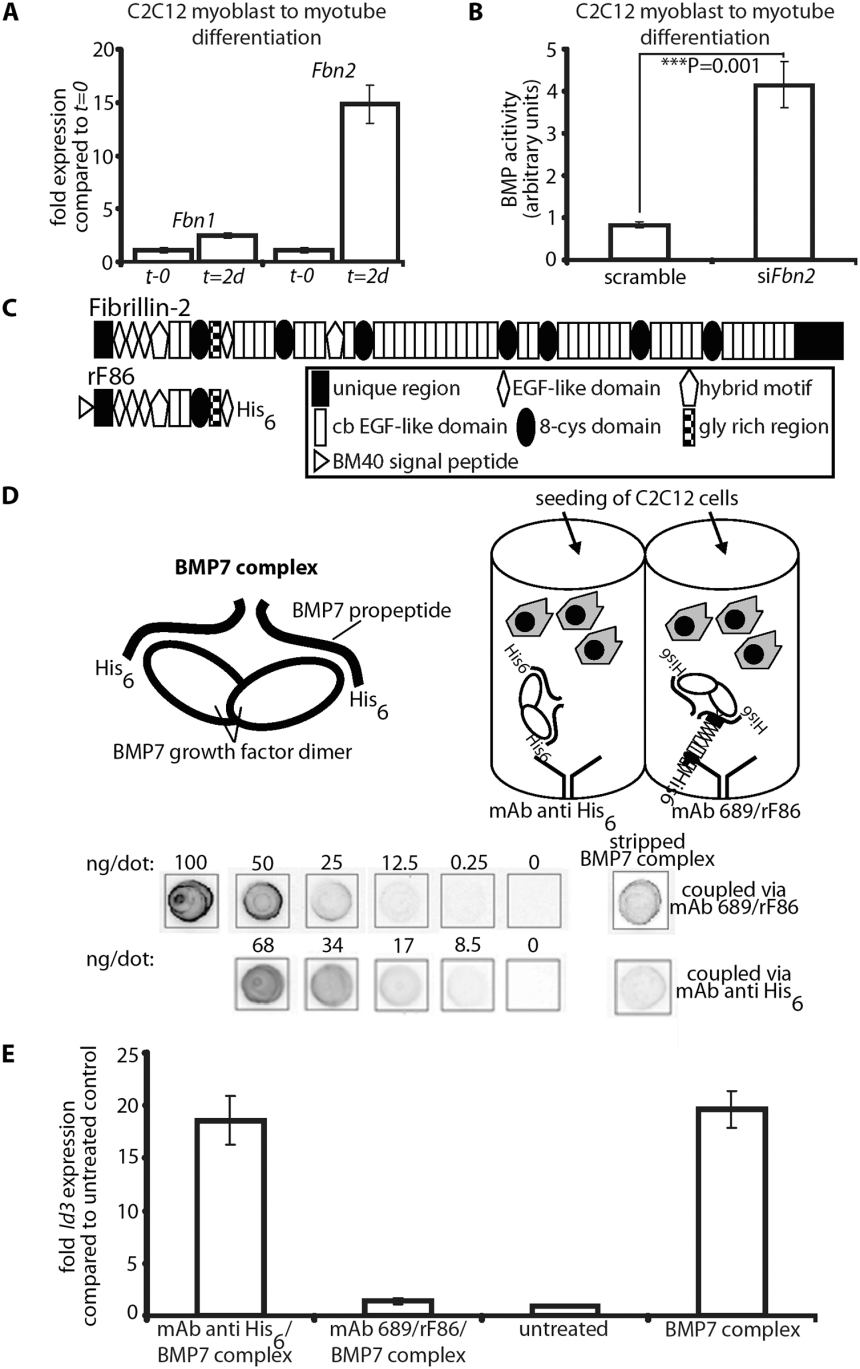
In vitro BMP bioactivity assay using C2C12 cells. **(A)** Comparison of *Fbn1* and *Fbn2* mRNA levels during myoblast to myotube differentiation. **(B)** BMP activity measured with and without RNAi of *Fbn2* during myoblast to myotube differentiation. **(C)** Domain structure of fibrillin-2 and recombinant polypeptide rF86. **(D)** Interaction of fibrillin-2 with the prodomain of BMP-7 complex confers latency to the growth factor. **Top left:** cartoon depicting BMP-7 complex, a growth factor dimer non-covalently associated with two processed prodomains. **Top right:** cartoon of the assay: BMP-7 complex was coupled via prodomain interactions either through a mAb directed against the N-terminal His_6_-tag of the BMP-7 prodomain or an N-terminal fibrillin-2 peptide (rF86) which was captured by mAb689. C2C12 cells were then seeded. **Below:** quantitation of coupled amounts of immobilized BMP-7 complex determined by comparison with standards of known concentration. **(E)** qPCR expression levels of *Id3* from treated and untreated wells. Error bars indicate mean ± SD. Each experiment was performed in triplicates.

To gain further insight into the molecular mechanisms by which fibrillin-2 might control BMP activity, we performed *in vitro* experiments. First, we measured the mRNA expression levels of *Fbn1* and *Fbn2* during mouse C2C12 myoblast to myotube differentiation. *Fbn2* mRNA was 15-fold increased 2 days after initiation of differentiation when compared to levels at the initiation start, while *Fbn1* mRNA expression was increased only 2.5-fold (Fig 8A). In a second experiment, we performed the C2C12 myoblast to myotube differentiation assay while *Fbn2* expression was knocked down by RNAi. Two days after initiation of differentiation the cell culture supernatant was removed and added to C2C12BRA cells, which harbor the BMP-inducible plasmid BRE-luc and serve as reporter cells for BMP bioactivity [28]. Supernatant from cells subjected to *Fbn2* RNAi contained 4 times more BMP bioactivity than supernatant from control cells (Fig 8B). These results were consistent with *in vivo* observations of increased BMP activity in *Fbn2* null forelimb muscle. In addition, the *in vitro* results indicate that, in the process of muscle maturation, fibrillin-2 performs a much more predominant role than fibrillin-1.

Based on the data, it seemed likely that fibrillin-2 is a negative regulator of BMP activity in muscle. We therefore hypothesized that binding to fibrillin-2 might confer latency to the BMP complex. To test this hypothesis, we created an assay using different methods for presenting BMP-7 complex to C2C12 cells. The positive control condition was addition of BMP-7 complex to the medium (BMP-7 complex in solution). To immobilize BMP-7, we tested 3 different substrates adsorbed onto plastic wells: (1) rF86, an N-terminal recombinant polypeptide of fibrillin-2 (Fig 8C); (2) an antibody (mAb 689) coupled to rF86 (Fig 8D); (3) a capture antibody against the N-terminal His_6_-tag of the BMP-7 prodomain (Fig 8D). The rF86 polypeptide was chosen because it displayed high affinity binding (K_d_ = 8–25 nM) in previous interaction studies with BMP prodomains [6,29]. We adsorbed mAb689, which we previously described as a pan-fibrillin antibody that recognizes a site close to EGF4 [9], to the well and then coupled rF86 to mab689. We then immobilized BMP-7 complex to the adsorbed substrates. To quantitate and compare the efficiency of BMP-7 immobilization through the two different capture approaches, the immobilized BMP-7 complex was stripped and compared to standards of known concentrations of BMP-7 complex in a dot blot immunoassay. mAb2, specific for the BMP-7 prodomain [5] was used for detection. Both capture approaches resulted in immobilization of quantitative amounts of BMP-7 complex, although rF86 (coupled to mAb689) captured 3-fold more BMP-7 complex than the anti-His_6_-tag antibody.

In order to measure BMP bioactivity, C2C12 cells were seeded onto the immobilized BMP-7 complexes captured by the two different approaches. After 6 hours, the total mRNA was immediately harvested, and mRNA expression levels of *Id3*, a BMP responsive element, were monitored by qPCR. BMP-7 complex simply added to the medium and BSA coated wells (“untreated”) served as positive and negative controls. Immobilizing 1.7 ng/well of BMP-7 complex, using the His_6_-tag, resulted in 18-fold induction of *Id3*, which was equivalent to adding 30 ng/well BMP-7 complex to the medium (Fig 8E). This demonstrated a more than 15-fold increase of BMP-7 activity when BMP-7 is localized to a scaffold at the bottom of the well compared to BMP-7 in solution. However, immobilizing BMP-7 complex through rF86 bound to the dish reduced BMP bioactivity to negative control levels. Binding of BMP-7 complex to fibrillin-2 followed by stripping off BMP-7 did not result in any reduction of BMP-7 bioactivity (S8 Fig), indicating that BMP-7 is reversibly inactivated by binding to fibrillin-2.

## Discussion

Collectively, our investigations demonstrate that the absence of fibrillin-2 causes a delay in forelimb muscle differentiation, contractures that resolve as muscle architecture improves, and infiltration of fat into the limb connective tissue space. Neonatal contractures in fibrillin-2 null mice were associated with a delay in the appearance of a specific perinatal myosin heavy chain. Mutations in the gene (*MYH8*) encoding this perinatal myosin heavy chain cause a human syndrome characterized by distal contractures, indicating that contractures in fibrillin-2 null mice may be the result of effects on myosin heavy chain-8. *In vitro* and *in vivo* experiments showed that abnormal activation of BMP signaling was the likely reason for the delay in muscle differentiation, including the delay in expression of myosin heavy chain -8, and for fatty infiltration of the limb connective tissue.

The finding that activation of BMP signaling caused decreased muscle mass in *Fbn2* null mice is surprising, since genetic ablation of Smad4 in adult mice caused muscle atrophy and weakness in response to denervation, while overexpression of an activated form of Alk3 (a type I BMP receptor) could rescue the *Smad4* muscle phenotype and overexpression of noggin (an inhibitor of BMP signaling) caused muscle hypertrophy [20]. Similarly, when BMP-7 was overexpressed in muscle, muscle mass was increased along with muscle hypertrophy [21]. The major difference between these studies and ours is that, in our study, activated BMP signaling is the result of a deficient environment and not the result of overexpression of a BMP ligand or inhibitor. Furthermore, our study focuses on very early postnatal muscle development, when fibrillin-2 performs an important role, whereas these other studies [20,21] examine the role of BMP signaling in adult mice, particularly in response to denervation. When *Bmpr1a* (*Alk3*) was knocked out in *Myf5 Cre* expressing cells, mice were born runted and muscle mass was reduced, but muscles appeared unaffected when *MyoD Cre* was used to knockout *Bmpr1a* [22]. These latter results are consistent with the concept that BMP signaling may exert different effects in different microenvironments. In contrast to these studies that directly manipulate known components of the BMP signaling pathway, our study shows the exquisite sensitivity of BMP signaling to the fibrillin-2 extracellular environment during early postnatal muscle development.

TGFβ signaling is known to be context-dependent. TGFβ has been described as a “cellular switch” that provides “a mechanism for coupling a cell to its environment” [30]. Results presented here indicate that fibrillin-2 is an important constituent of the skeletal muscle environment and that BMP signaling is coupled to the fibrillin-2 microenvironment. Previously, syndactyly in *Fbn2* null mice was associated genetically with a loss of BMP signaling [11]. In the studies presented here, loss of fibrillin-2 in the interdigital space was accompanied by a loss of fibrillin-1 (S6 Fig), but in P0 skeletal muscle, fibrillin-1 seemed grossly similar to wildtype in the absence of fibrillin-2 (S7 Fig). More information is required in order to know why loss of fibrillin-2 in the early postnatal skeletal muscle environment leads to activation of BMP signaling.

As a first step, we addressed whether direct binding to fibrillin-2 can regulate BMP signaling. In previous studies, we demonstrated that BMP growth factors form complexes with their prodomains and that the prodomain targets the prodomain/growth factor complex to specific sites on fibrillins [5,6,29]. We also showed that some BMP complexes (BMP-4, -5, and -7) are as bioactive in solution as the free BMP growth factor dimer [6,29], suggesting that unlike the prodomains of TGFβ, GDF8, BMP-10, and other members of the TGFβ superfamily [29,31–33], BMP prodomains do not perform an intrinsic inhibitory role [34]. Recently, the crystal structure of BMP-9 prodomain/growth factor complex provided a molecular description of active BMP complexes such as BMP-9 and BMP-7: these complexes share an “open” conformation” [35], unlike the TGFβ small latent complex, which is constrained by the propeptides into a more closed conformation [36].

Here in this study, we found that, when BMP-7 complex is targeted to a solid substrate (by mAb recognizing the His_6_-tag on the prodomain) close to cells, it is potentiated. We interpret these results to suggest that spatial concentration of BMPs to a protein scaffold close to cells dramatically increases their bioactivity when compared to the same numbers of freely diffusing BMP molecules. In addition, we showed that, when BMP-7 complexes are bound to a fibrillin-2 scaffold, BMP bioactivity was inhibited. This inhibition by fibrillin binding is most likely induced by a conformational change in the prodomain structure that prevents access of BMP receptors to the growth factor. Interestingly, molecular modeling indicated that BMP-9 prodomain/growth factor complex can adopt a closed conformation similar to the TGFβ small latent complex [35]. Our ongoing studies are addressing whether binding to fibrillin can induce such a conformational change in BMP-7 complex.

Our new mechanistic insights into the control of BMP bioactivity by fibrillins have important implications: if BMP complexes are latent when bound to fibrillin, then there must be mechanisms for activating BMP signaling in appropriate cellular contexts. These mechanisms are unknown, since it has been thought that BMPs do not require activation. It is possible that cells may activate fibrillin-bound BMP complexes through mechanical means, either pulling on the prodomain, as in the case of integrin pulling to activate latent TGFβ [36] or pulling on fibrillin itself to locally release BMP complexes close to cellular BMP receptors. This activation mechanism may be differentially utilized by cells, depending on their expression of appropriate cellular receptors. We currently do not know what cellular receptors may activate BMP complexes bound to fibrillin.

Although we cannot currently explain why BMP signaling is activated in the skeletal muscle of fibrillin-2 null mice, we can speculate. One possible explanation is that, in the absence of fibrillin-2, BMP complexes are not bound by fibrillin-2 and therefore not inhibited. However, our *in vitro* experiment suggests that free, diffusing BMP complexes would be much less potent than the same quantities of targeted BMPs. A second possibility is that, in the absence of fibrillin-2, BMP complexes are mistargeted to fibrillin-1. On fibrillin-1, without fibrillin-2 present, BMP complexes may be more concentrated than usual and potentially more potent. The conformation of fibrillin-1 microfibrils, in the absence of fibrillin-1, may lead to easier activation of BMPs, particularly during the early postnatal period when we were able to detect differences in resistance to pepsin. Further maturation and stabilization of fibrillin-1 microfibril conformation during the first week of postnatal life would be consistent with this explanation, since activation of BMP signaling is limited to the first few days after birth. In addition, fibrillin-1 may occupy specific sites, vis a vis certain muscle cells, distinct from those occupied by fibrillin-2. Future investigations will be directed toward elucidating the spatially specific muscle cell niches defined by fibrillin-1 compared to fibrillin-2.

Our results can be taken in several translational directions. First, the myopathy caused by the absence of fibrillin-2 in mice may be relevant to human congenital muscular dystrophies. In contrast to heterozygous mutations in *Fbn2* causing CCA, with contractures and muscle weakness, results in mice predict that homozygous *Fbn2* mutations may cause a form of human congenital muscular dystrophy. In humans, an autosomal recessive form of congenital muscular dystrophy with arthrogryposis (OMIM %253900) has been reported. The most recent report [37] described a family in which a child died of respiratory failure two hours after birth. Although the lungs were normal, the skeletal muscle was replaced by adipose and some connective tissue. Skeletal muscle from another fetus, electively terminated when no movements were detected after 19 weeks of gestation, showed multiple contractures and severe skeletal muscle wasting with adipose tissue replacement. *Fbn2* null mice on the C57Bl/6 background may model this type of congenital muscular dystrophy: death just after birth, contractures, skeletal muscle wasting with adipose tissue replacement, normal lungs, and autosomal recessive inheritance.

A second translational direction is the potential for identification of genetic modifiers of congenital muscular dystrophy. *LTBP4* has been identified as an important genetic modifier of muscular dystrophy in mice [38] and in boys with DMD [39]. It is interesting to note here that LTBP-4 is a fibrillin-like molecule that is incorporated into fibrillin microfibrils. Similar approaches could be used to identify the genetic modifier(s) in 129 vs. C57/Bl6 mice controlling severity of the *Fbn2* myopathy. These genetic modifiers could then be tested to determine utility in the clinic to predict severity of common forms of muscular dystrophy.

Third, since fibrillin microfibrils integrate basement membrane molecules with the adjacent connective tissue, it is possible that defects in cell-matrix interactions that underlie congenital muscular dystrophies may perturb fibrillin microfibrils and cause dysregulation of BMP signaling. Adenoviral overexpression of Noggin in the muscle of *mdx* mice (a model of DMD) resulted in a reduction in fibrotic/necrotic area and an improvement in muscle histology [40], indicating that abnormally activated BMP signaling may contribute to DMD and perhaps to other congenital forms of muscular dystrophy. However, mechanisms underlying this improvement of *mdx* muscle were not investigated. Our new results provide a potential mechanism by which BMP signaling may be activated in some forms of congenital muscular dystrophy, if perturbation of fibrillin microfibrils is a general or specific consequence of defects in muscle cell-matrix interactions.

Fourth, a better understanding of the molecular mechanisms by which fibrillins control BMP signaling is required before therapeutics can be designed to specifically target the muscle cell microenvironment. We have shown that the absence of fibrillin-2 has tissue-specific effects on the skeletal muscle, but not on other tissues. If activated BMP signaling contributes to myopathy and infiltrating fat, then modulating the fibrillin-2/BMP interaction may be a clever therapeutic strategy.

Lastly, our results advance knowledge regarding potential cellular mechanisms by which muscle is replaced with fat, a key clinical feature of muscular dystrophy. Here we showed that active BMP-7 signaling performs a critical role in the formation of white fat. BMP-7 is known to play a role in the induction of brown adipocytes from progenitor cells residing within skeletal muscle [41,42]. In contrast to these primarily *in vitro* studies, our results demonstrated that ablation of one allele of *Bmp7* is sufficient to rescue the induction of white fat in *Fbn2* null skeletal muscle, demonstrating an *in vivo* role for BMP-7 in the transdifferentiation of cells from muscle to white fat. Whether this role is specific for BMP-7 is currently under investigation. Genetic approaches will be used in the future to reveal the identity of the BMP-7 responsive cells that are induced to become white adipocytes.

## Materials and Methods

### Mice

*Fbn2* null mice [11] were maintained on a 129/Sv background and genotyping by PCR was performed as previously described [3]. *Bmp7* ^*+/-*^ mice were maintained on a 129S/SvEv-Gpi1^c^ background. For genotyping, wildtype and mutant *Bmp7* alleles were amplified by PCR as previously described [27]. *Fbn-* ^/-^ mice were bred to *Bmp7* ^+/-^ mice to yield *Fbn2* ^+/-^; *Bmp7* ^+/-^ mice. Doubly heterozygous mutant mice were bred to each other to generate *Fbn2* ^-/-^; *Bmp7* ^+/-^, and all progeny were genotyped by PCR. *Fbn2* ^*+/-*^ mice on 129/Sv background were bred to wildtype C57/Bl6 mice, and *Fbn2* ^*+/-*^ progeny were bred to wildtype C57/Bl6 mice for nine generations. *Fbn2* ^*+/-*^ mice were bred to each other to generate *Fbn2* ^*-/-*^ mice after six and nine generations of backcrossing. Maintenance of mice and all experimental procedures were conducted in accordance with National Institutes of Health guidelines and were approved by the OHSU IACUC (approval number IS00003821).

### Histopathology and immunofluorescence microscopy

Serial 4 μm cryosections of frozen muscle were prepared and stained with hematoxylin and eosin (H&E) using the Shandon Rapid Chrome H&E frozen section staining kit (Thermo Scientific, Waltham, MA) according to the manufacturer’s protocol. Sections were examined using a Zeiss Axiophot microscope, and micrographs were recorded digitally using AxioVision software (version 4.5; Zeiss, Germany). In addition, fresh tissues were fixed in 1.5% glutaraldehyde/1.5% paraformaldehyde with 0.05% tannic acid in cacodylate buffer, followed by 1% buffered OsO_4_ and then rinsed, dehydrated, and embedded in Spurr’s epoxy. Thick sections (8 μm) from these strong-fixed tissue blocs were stained with Toluidine blue O stain (Sigma, St. Louis, MO) and examined by light and electron microscopy.

Immunofluorescence microscopy was performed as previously described [3,43]. Primary antibodies, polyclonal rabbit anti-phospho Smad1/5/8 (9511, Cell Signaling, Danvers, MA) or anti-phospho Smad2/3 (3101, Cell Signaling), and secondary antibodies, Alexa 488 goat anti-rabbit or Alexa 568 goat anti mouse (Invitrogen Molecular Probes, Eugene, OR), were used at a concentration of 1:1000.

### Creatine kinase activity assay

Citrated plasma was stored at -80°C until used. Plasma was assayed using a colorimetric Creatine Kinase Activity Assay Kit, according to the manufacturer’s protocol (Abcam, Cambridge, MA). Briefly, in a 96-well plate an NADH standard curve was developed (0–10nmol), along with blood samples. 5 μl of sample were brought to 50 μl with addition of assay buffer. 50 μl of a reaction mix containing assay buffer, enzyme mix, developer, ATP, and substrate was added to each well. The plate was incubated at 37°C for 40 minutes with spectrophotometric readings at 450 nm taken every 2 minutes. Each sample was tested in one well of a 96-well plate. Multiple dilutions of each sample were tested to ensure that readings were within the standard curve range. Creatine kinase activity was calculated by the equation: (nmol NADH/(reaction time X sample volume in ml)) X dilution factor = nmol/min/ml.

### Pepsin extraction of muscle

Pepsin digests of mouse P0-P8 forearm muscle was performed as previously described [24]. In brief, total forearm muscle was dissected, dropped in liquid nitrogen, and ground with a mortar and pestle. The pulverized tissue was first washed with cold water, then three times with cold 1 M NaCl (20 μl per mg tissue), and once more with water. The final pellet was resuspended in 0.5 M acetic acid and the stirred suspension was digested with pepsin (Sigma; 8 μg of pepsin/1 mg wet weight) for 16 h at 4°C. The solubilized material was collected following centrifugation. Sodium chloride (10%, w/v) was added to the supernatant, and the precipitate was collected by centrifugation. The pellet was resuspended in 0.1 M Tris-HCI, pH 8.1, and stirred at 4°C for at least 72 hours to inactivate the pepsin. Equal protein amounts extracted from *Fbn2* ^+/+^, *Fbn2* ^+/-^, and *Fbn2* ^-/-^ muscle (determined by using the Pierce BCA protein assay kit (Pierce, Rockford, IL)) were loaded onto a 4–20% polyacrylamide gel and analyzed under non-reducing conditions, followed by Western blotting using pAb 9543, specific for fibrillin-1. For quantitation of the Myh8 protein band, Ponceau S stained membranes were scanned, and band intensities were measured using NIH ImageJ (Rasband); results were normalized relative to the pepsin resistant collagen band. Results shown were generated from N = 3 litters containing littermates of each genotype for P1, and N = 2 litters for later time points.

### Myoblast to myotube differentiation assay and RNAi

Mouse C2C12 cells (CRL-1772, ATCC, Manasassas, VA) were cultured in Dulbecco’s modified Eagle’s medium (DMEM) (MediaTech, Hendon, VA) supplemented with 10% fetal bovine serum (Atlanta Biologicals, Lawrenceville, GA) and penicillin/streptomycin (MediaTech). Cells were seeded on Costar 6 well plates (Sigma) at 50,000 cells/well and transfected at 30% and 60% confluency with 100 μM *Fbn2* siRNA and scrambled control using Lipofectamine reagent according to the manufacturer’s instructions (all reagents from Invitrogen). At 90% confluency, cells were stimulated with DMEM containing 2% horse serum for 48 hours. The cell culture supernatant was analyzed for BMP activity by pipetting it onto stably transfected cells carrying BMP-responsive elements from the *Id1* promoter fused to a luciferase reporter gene, as previously described [28]. Total RNA of the cell layer was harvested using 1 ml TRIzol reagent (Invitrogen), and expression of myogenic markers was monitored by real-time quantitative PCR.

### Real-time quantitative PCR (qRT-PCR)

1–10 mg of dissected P0 or P8 mouse forearm muscle was dropped into 0.8 ml of TRIzol reagent (Invitrogen, Carlsbad, CA) and ground with a micropestle (Kimble-Chase, Vineland, NJ) into smaller pieces. RNA extraction was performed according to the manufacturer’s protocol. A subsequent sample purification step was included using the RNeasy kit (Qiagen, Valencia, CA), and residual DNA contamination was removed from each sample by using the Turbo DNA-free kit (Ambion, Austin, TX). RNA samples were quantified by photospectrometry, and 0.1 to 1.0 μg of RNA per sample was reverse transcribed using the Bio-Rad iScript cDNA synthesis kit (Bio-Rad, Hercules, CA). Samples were amplified in triplicates using the iTaq SYBR Green Supermix (Bio-Rad) in an iQ5 Multicolor Real-Time PCR Detection System (Bio-Rad). Analysis of data was performed using the 2^-ΔΔCt^ method [44] and quantitated relative to the ARBP0 gene. Gene expression was normalized to littermate wildtype control mice, which provided an arbitrary constant for comparative fold expression. Primers used for qRT-PCR are listed in Table 2.

**Table 2:** Primers used for quantitative real-time PCR.

name	sequence	orientation
q*Bmp2*F	5’-caggaagctttgggaaacag-3’	forward
q*Bmp2*R	5’-tcgaagctctcccactgact-3’	reverse
q*Bmp4*F	5’-cagggcttccaccgtataaa-3’	forward
q*Bmp4*R	5’-cagggctcacatcgaaagtt-3’	reverse
q*Bmp5*F	5’-ttcaaggcaagcgaggtact-3’	forward
q*Bmp5*R	5’-tgcaggcttgtttttgttca-3’	reverse
q*Bmp6*F	5’-tctacaacgccctgtccaat-3’	forward
q*Bmp6*R	5’-aggagactcttgcggttcaa-3’	reverse
q*Bmp7*F	5’-ggaagcatgtaagggttcca-3’	forward
q*Bmp7*R	5’-ttcctggcagacatttttcc-3’	reverse
F*Mrf4*	5’-ctcagcctccagcagtcttc-3’	forward
R*Mrf4*	5’-gttccaaatgctggctgagt-3’	reverse
F*Myf5*	5’-aagctttcgagacgctcaag-3’	forward
R*Myf5*	5’-ttctccacctgttccctcag-3’	reverse
F*Myogenin*	5’-gaaagtgaatgaggccttcg-3’	forward
R*Myogenin*	5’-gagctgagcaaggcctgtag-3’	reverse
F*MyoD*	5’-aactgctctgatggcatgat-3’	forward
R*MyoD*	5’-tcgacacagccgcactcttc-3’	reverse

For quantitation of absolute BMP mRNA expression levels, 500 bp PCR fragments were generated which served as external standard templates for each of the BMP qPCR primer sets utilized. Real-time PCR reactions using a dilution series of known concentrations for each individual standard were simultaneously run next to reactions using reverse transcribed mRNAs from P0 mouse forearm muscles as templates. Standard curves were generated by graphing threshold cycles of standard PCR reactions against the concentrations of the standard templates. The absolute amounts of each BMP mRNA were then calculated by comparing threshold cycles obtained from reactions using reverse transcribed forearm RNAs as templates with the standard curves. Sequences of primers used to generate the 500 bp standard templates are listed in Table 3.

**Table 3:** Primers for generating template standards to absolutely quantitate BMP mRNA amounts.

name	sequence	orientation
m*Bmp*2stdF	5’-aagaagccgtggaggaactt-3’	forward
m*Bmp*2stdR	5’-tgacgcttttctcgtttgtg-3’	reverse
m*Bmp*4stdF	5’-aggaggaggaagagcagagc-3’	forward
m*Bmp*4stdR	5’-ccactcccttgaggtaacga-3’	reverse
m*Bmp*5stdF	5’-gggctggcttgtctttgata-3’	forward
m*Bmp*5stdR	5’-tggtctggaaacatcaggtg-3’	reverse
m*Bmp*6stdF	5’-ctcttcttcgggcttcctct-3’	forward
m*Bmp*6stdR	5’-tgtggggagaactccttgtc-3’	reverse
m*Bmp*7stdF	5’-tcgacgacagctctaatgtca-3’	forward
m*Bmp*7stdR	5’-ttcattcatgggttttattgtga-3’	reverse

### Limb organ culture

Limbs from neonatal mice were skinned, trimmed proximally and distally, washed 2 times in DMEM containing penicillin/streptomycin, and cultured for 48hs in DMEM with 10% fetal bovine serum containing either 200–800 ng mouse Noggin (R&D Systems, Minneapolis, MN) or 600–2400 ng BMP-7 complex [5] on a rocking platform. Untreated samples served as controls. Three independent organ culture experiments were carried out and analyzed. Each time, forelimbs from different animals were used. All animals were littermates.

### Micro-computed tomography

μCT analysis of epoxy embedded limbs was performed using a Scanco μCT 35 instrument (Scanco Medical, Basserdorf, Switzerland), according to the manufacturer’s instructions. Skinned forelimbs were fixed and treated with OsO_4_ as described [45]. Series of digital limb cross sections were generated by μCT. Areas of fat, muscle and bone were quantitated using NIH ImageJ (Rasband) and computed as percentages of the total area of the cross section.

### BMP bioactivity assay

mAb 689 (17.4 μg/ml in coating buffer) was adsorbed overnight onto all wells of an ELISA plate. 5% milk was incubated as a blocking solution for 1 hour. Then, the wells were washed three times with TBS/Tween and subsequently incubated with 0.5 μM rF86 in 2% milk for 2 hours. The plate was washed again and incubated with 47.6 μg/ml BMP-7 complex (0.5 μM) in 2% milk for 2 hours. After final washes, the plate was incubated with 100 μl/well of 0.1M Glycine, pH 2.3, for 20 minutes in order to strip off the bound BMP-7 complex. The contents of all 96 wells were pooled and dialyzed against 0.1M acetic acid overnight. This solution was lyophilized and resuspended in 50 μl TBS. 5 μl dots were placed on a nitrocellulose membrane together with a diluted series of dots containing BMP-7 complex at known concentrations (the standard curve). After drying, the membrane was blocked in 5% milk and incubated with anti-BMP-7 prodomain antibody mAb2 (1 mg/ml, 1:500) for 2 hours. The membrane was washed and subsequently incubated with an HRP-conjugated goat anti-mouse antibody for 2 hours. After the final washes, signals were developed using the Bio-Rad Opti 4CN Substrate kit. The membrane was scanned and signals were quantitated using image quant software. A similar procedure was performed after first adsorbing an anti-His_6_-tag mAb onto all wells of an ELISA plate.

C2C12 cells were seeded onto the plates in which quantitated amounts of BMP-7 complex had been immobilized. After 6 hours, the total mRNA was immediately harvested, and mRNA expression levels of *Id3* were monitored by qPCR. BMP-7 complex was added to the medium to serve as a positive control, and BSA coated wells served as negative controls.

### Statistical analysis

Statistical analysis of muscle mass measurements, myofibril counts on H&E stained sections, and muscle and fat quantitations in P8 muscles were generated with GraphPad Prism 5.0 for Windows (GraphPad, San Diego, CA). P values were obtained from a One-way Analysis of Variance (1-way ANOVA) with Significance level Alpha = 0.05.

## Supporting Information

S1 Fig Hindlimb muscle and fat in P5 wildtype and *Fbn2* null mice. 3 μm micro-CT digital sections through comparable hindlimb regions of *Fbn2* ^*+/+*^ and *Fbn2* ^*-/-*^ mice are shown (left). Bars = 1 mm. The areas of fat and muscle were quantitated using Image J, and calculated as percentages of the total area of the cross section. Single animals are shown. Results suggest that *Fbn2* null hindlimbs also show a reduction in muscle and an increase in fat.
(TIF)

S2 Fig Expression levels of *Fbn1* and *Fbn2* in Fbn2 heterozygous and null forelimbs. Forelimb muscles from animals from four different litters (n = 4), each containing wildtype, *Fbn2* ^*+/-*^, and *Fbn2* ^*-/-*^ mice, were analyzed using two different *Fbn1* and *Fbn2* qPCR primer sets. The values reflect fold changes over wildtype littermate controls. As expected, *Fbn2* ^*+/-*^ muscles show a 50% reduction in expression of *Fbn2*, and *Fbn2* ^*-/-*^ muscles show no expression of *Fbn2*. *Fbn1* levels were not affected by genetic ablation of *Fbn2*.
(TIF)

S3 Fig Quantitation of Ponceau S-stained membranes with pepsin digests of forelimb muscle. Similar to the membrane shown in Fig 4A, additional membranes are shown (top). Three litters containing all genotypes were used for P1, and two litters containing all genotypes were used for P3. Membranes were scanned and specific band intensities were quantitated using Image J. The Myh8 protein band intensities were normalized to the pepsin resistant collagen bands. Myh8 protein amounts were significantly reduced from P1-P3.
(TIF)

S4 Fig qPCR of *Fbn1* expression in C2C12 cells treated with scrambled control or with *Fbn2* siRNA. No differences were found in expression levels of *Fbn1* between cells treated with *Fbn2* siRNA compared to scrambled control. Results were obtained from three independent experiments, and each experiment was performed in triplicates. Error bars indicate mean ± SD.
(TIF)

S5 Fig qPCR of *Id1* expression in P0 wildtype, heterozygous and *Fbn2* null forelimbs. Three animals of each genotype were utilized. When compared to wildtype, expression of *Id1* in *Fbn2* null forelimb muscle showed a statistically significant increase in expression.
(TIF)

S6 Fig Whole-mount confocal microscopy of E13.5 mouse autopods. Wildtype autopods (top) or fibrillin-2 null autopods stained with antibodies to fibrillin-1 or fibrillin-2. Digits (asterisk) and interdigital space (arrows) are marked. Bars = 80 μm.
(TIF)

S7 Fig Immunofluorescence of P0 wildtype and *Fbn2* null muscle. Sections were double labeled with antibodies specific for Pax-7 (red) and fibrillin-1 or fibrillin-2 (green). Arrows indicate fibrillin microfibrils in close proximity to satellite progenitor cells positive for Pax-7. Bars = 20 μm.
(TIF)

S8 Fig BMP bioactivity after binding of fibrillin-2 to the BMP-7 prodomain. qPCR of *Id3* showed that the BMP-7 growth factor is not irreversibly inactivated by binding to fibrillin-2. Error bars indicate mean ± SD. Each experiment was performed in triplicates.
(TIF)

S1 Text Supplementary materials contains additional methods used for generation of supplementary figures. (DOCX)
