# Supplementary figures and images for: Abnormal Activation of BMP Signaling Causes Myopathy in Fbn2 Null Mice
Source: PLoS Genet. 2015 Jun 26;11(6):e1005340. doi: 10.1371/journal.pgen.1005340 (PMC4482570; doi:10.1371/journal.pgen.1005340)

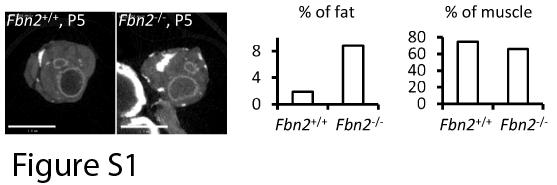

Supplement: S1 Fig — 3 μm micro-CT digital sections through comparable hindlimb regions of Fbn2 +/+ and Fbn2 -/- mice are shown (left). Bars = 1 mm. The areas of fat and muscle were quantitated using Image J, and calculated as percentages of the total area of the cross section. Single animals are shown. Results suggest that Fbn2 null hindlimbs also show a reduction in muscle and an increase in fat. (TIF) [file pgen.1005340.s001.tif]

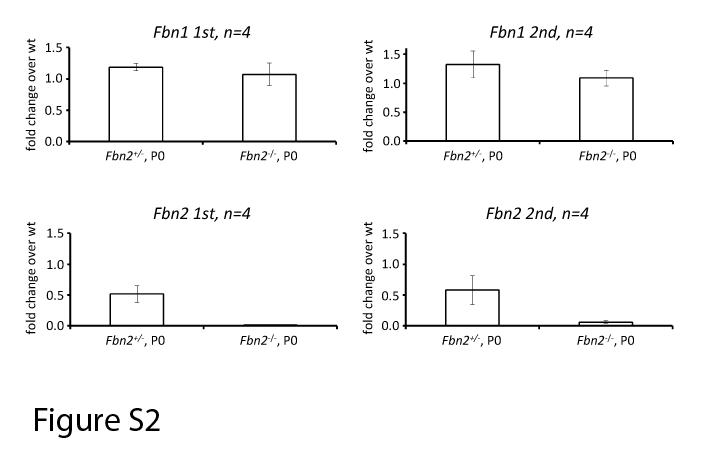

Supplement: S2 Fig — Forelimb muscles from animals from four different litters (n = 4), each containing wildtype, Fbn2 +/-, and Fbn2 -/- mice, were analyzed using two different Fbn1 and Fbn2 qPCR primer sets. The values reflect fold changes over wildtype littermate controls. As expected, Fbn2 +/- muscles show a 50% reduction in expression of Fbn2, and Fbn2 -/- muscles show no expression of Fbn2. Fbn1 levels were not affected by genetic ablation of Fbn2. (TIF) [file pgen.1005340.s002.tif]

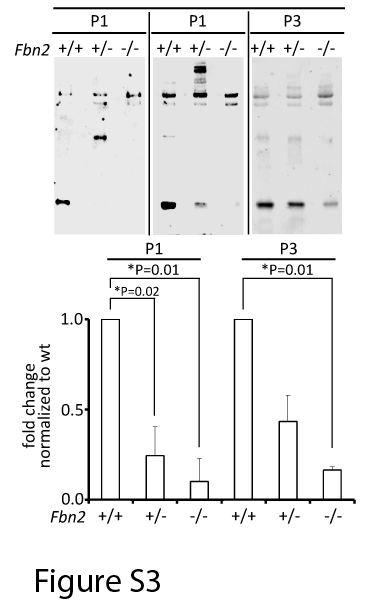

Supplement: S3 Fig — Similar to the membrane shown in Fig 4A, additional membranes are shown (top). Three litters containing all genotypes were used for P1, and two litters containing all genotypes were used for P3. Membranes were scanned and specific band intensities were quantitated using Image J. The Myh8 protein band intensities were normalized to the pepsin resistant collagen bands. Myh8 protein amounts were significantly reduced from P1-P3. (TIF) [file pgen.1005340.s003.tif]

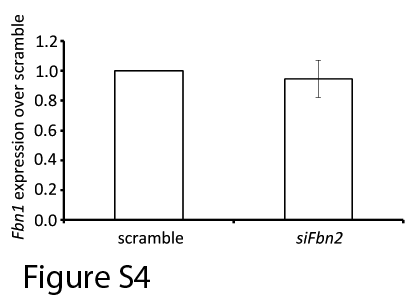

Supplement: S4 Fig — No differences were found in expression levels of Fbn1 between cells treated with Fbn2 siRNA compared to scrambled control. Results were obtained from three independent experiments, and each experiment was performed in triplicates. Error bars indicate mean ± SD. (TIF) [file pgen.1005340.s004.tif]

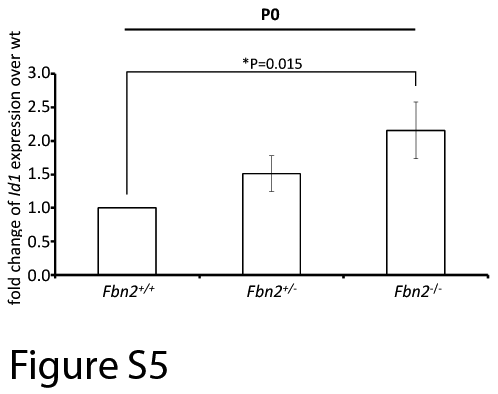

Supplement: S5 Fig — Three animals of each genotype were utilized. When compared to wildtype, expression of Id1 in Fbn2 null forelimb muscle showed a statistically significant increase in expression. (TIF) [file pgen.1005340.s005.tif]

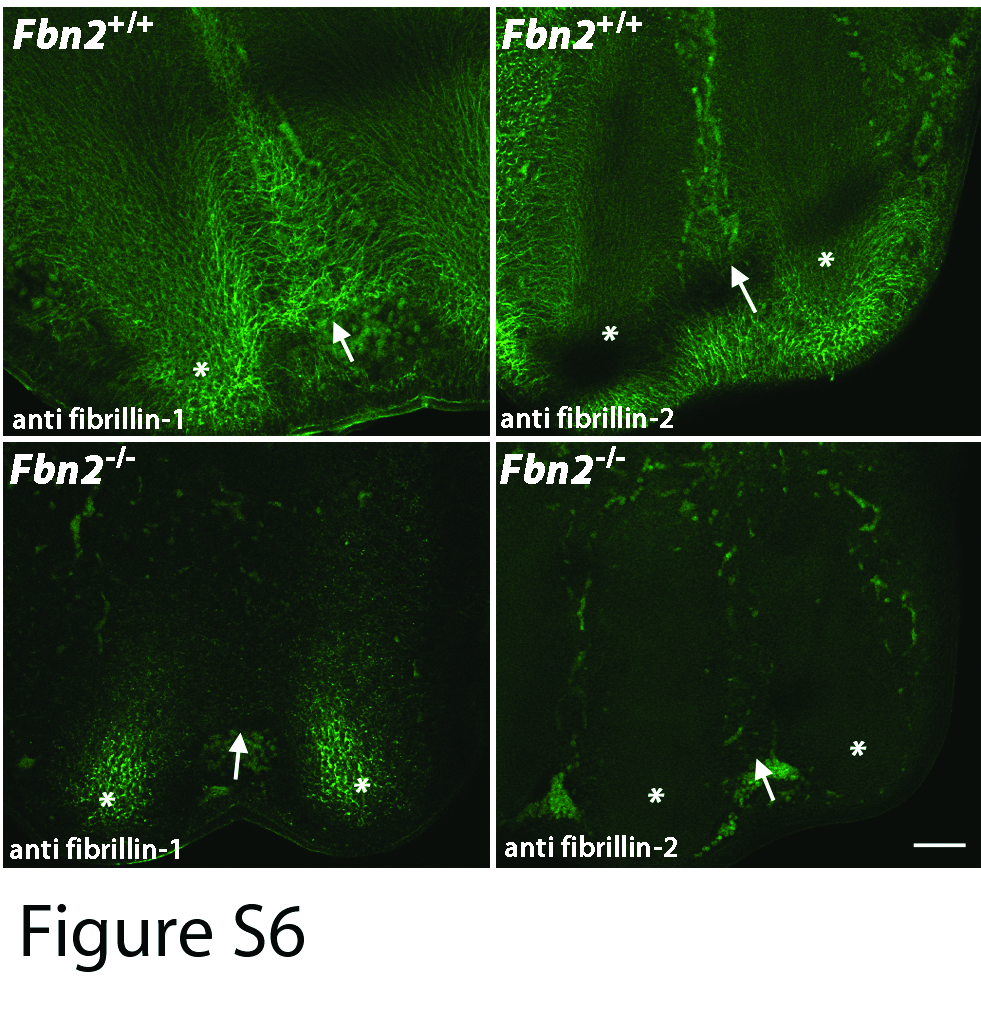

Supplement: S6 Fig — Wildtype autopods (top) or fibrillin-2 null autopods stained with antibodies to fibrillin-1 or fibrillin-2. Digits (asterisk) and interdigital space (arrows) are marked. Bars = 80 μm. (TIF) [file pgen.1005340.s006.tif]

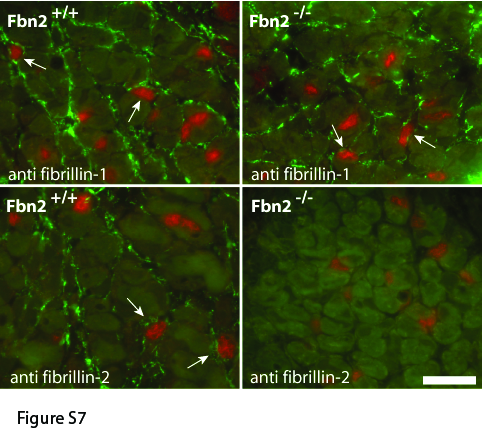

Supplement: S7 Fig — Sections were double labeled with antibodies specific for Pax-7 (red) and fibrillin-1 or fibrillin-2 (green). Arrows indicate fibrillin microfibrils in close proximity to satellite progenitor cells positive for Pax-7. Bars = 20 μm. (TIF) [file pgen.1005340.s007.tif]

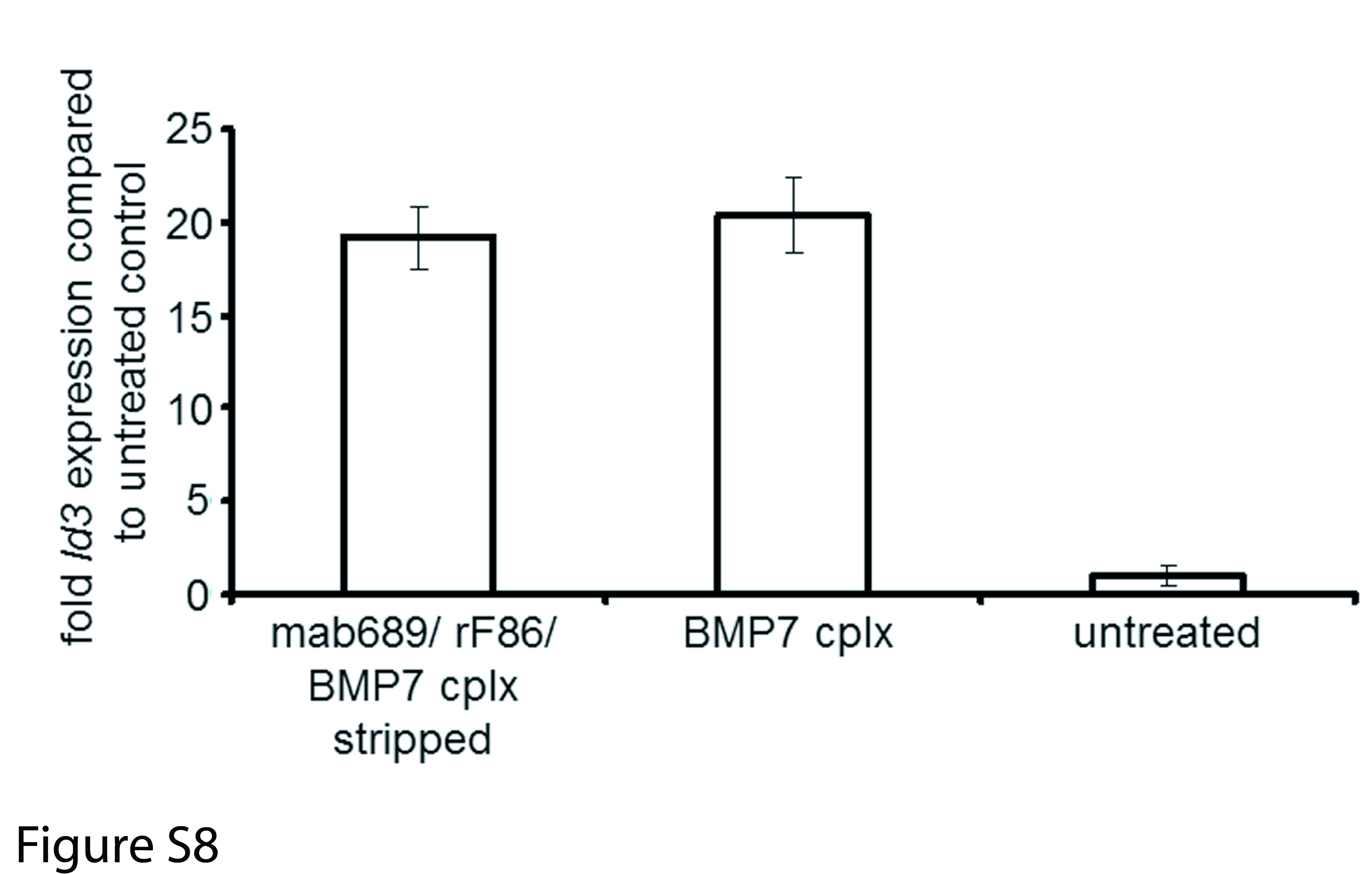

Supplement: S8 Fig — qPCR of Id3 showed that the BMP-7 growth factor is not irreversibly inactivated by binding to fibrillin-2. Error bars indicate mean ± SD. Each experiment was performed in triplicates. (TIF) [file pgen.1005340.s008.tif]
