# Supplementary material for: Abnormal Activation of BMP Signaling Causes Myopathy in Fbn2 Null Mice
Source: PLoS Genet. 2015 Jun 26;11(6):e1005340. doi: 10.1371/journal.pgen.1005340 (PMC4482570; doi:10.1371/journal.pgen.1005340)
Supplement: S1 Text — (DOCX) [file pgen.1005340.s009.docx]

**S1 Text: Supplementary Materials**

Materials and Methods

*Whole-mount confocal immunolocalization:*

Wildtype and *Fbn2* null E 13.5 embryos were collected and autopods were fixed in 20% DMSO/MeOH overnight at 4^o^. The autopods were washed with multiple changes of PBS over 1 hour at room temperature and incubated on a rocking platform overnight at 4^o^ with affinity-purified antibodies (pAb 9543, specific for fibrillin-1, and pAb 0868, specific for fibrillin-2), diluted 1:50 in PBS. Samples were then washed over 7 hours at room temperature with multiple changes of PBS. Autopods were incubated with secondary goat anti-rabbit Alexa 488 (Invitrogen), diluted 1:1000 in PBS, overnight at 4^o^ on a rocking platform. After final washes with several changes of PBS over 4.5 hours at room temperature, autopods were superglued onto cavity slides and dehydrates through several changes of MeOH. After clearing with benzyl alcohol and benzyl benzoate (1:2 v/v) (Murray’s Clear), samples were viewed and photographed using a Leica confocal microscope.

*Double immunofluorescence of fibrillins and Pax-7:*

For immunofluorescence analysis of fibrillin-1 and -2 together with Pax7, an epitope retrieval protocol was applied. In brief, 5 μm sections of previously snap frozen tissue were air dried overnight, fixed in 2% PFA, rinsed in PBS, followed by permeabilization in 0.1% TritonX-100/PBS. Subsequently, the slides were boiled for 1minute by microwaving at full power in 10mM citrate buffer at pH 6. After cooling and rinsing in PBS, slides were blocked in 10% normal goat serum containing 3% BSA in 0.1% TritonX-100/ PBS for 1hour at room temperature. Primary antibodies were diluted in blocking buffer over night at 4ºC at the following concentrations: polyclonal antibody (pAb) 9543 (anti-fibrillin-1) at 7 μg/ml, pAb868 (anti-fibrillin-2) at 5 μg/ml, and monoclonal antibody (mAb) 1675 (Pax7, R&D Systems) at 8 μg/ml.

*Bioactivity assay after binding to fibrillin-2:*

BMP7 complex was coupled via rF86, which was captured by mab689 on wells of an ELISA plate. Subsequently, wells were incubated with 100μl of 0.1M acetic acid for 1h at room temperature to strip the bound BMP7 growth factor dimer from this assembly. The stripped content of 6 wells (30ng) was combined and lyophilized. The resulting pellet was resuspended in PBS and added to C2C12 cells for 6 hours in a volume of 100μl serum free media. For comparison, C2C12 cells were incubated with 30 ng/well of fresh BMP-7 complex under the same conditions. As a readout for bioactivity, the expression of *Id3* (a BMP responsive element) was measured via qPCR.
